# Gene dependence during mammalian Acinetobacter baumannii pneumonia and septicaemia infections

**DOI:** 10.1099/mgen.0.001556

**Published:** 2025-11-11

**Authors:** Faye C. Morris, Francesca Short, Xenia Kostoulias, Cara Nethercott, Ying Fu, Yan Jiang, Thomas Smallman, Yusong Yu, Ian T. Paulsen, John D. Boyce, Anton Y Peleg

**Affiliations:** 1 Infection Program, Monash Biomedicine Discovery Institute, Department of Microbiology, Monash University, Clayton, Victoria, Australia; 2 Centre to Impact AMR, Monash University, Clayton, Victoria, Australia; 3 Department of Molecular Sciences, Faculty of Science and Engineering, Macquarie University, Sydney, New South Wales, Australia; 4 Department of Infectious Diseases, The Alfred Hospital and School of Translational Medicine, Monash University, Melbourne, Victoria, Australia; 5 Department of Clinical Laboratory, Sir Run Run Shaw Hospital, College of Medicine, Zhejiang University, Hangzhou, Zhejiang Province, PR China; 6 Key Laboratory of Precision Medicine in Diagnosis and Monitoring Research of Zhejiang Province, Hangzhou, Zhejiang Province, PR China; 7 Department of Infectious Diseases, Sir Run Run Shaw Hospital, College of Medicine, Zhejiang University, Hangzhou, Zhejiang Province, PR China; 8 Regional Medical Centre for National Institute of Respiratory Diseases, Sir Run Run Shaw Hospital, School of Medicine, Zhejiang University, Hangzhou, PR China

**Keywords:** *Acinetobacter baumannii*, functional genomics, pneumonia, septicaemia, tissue tropism, Tn-Seq

## Abstract

With a limited number of traditional virulence factors, the success of the nosocomial pathogen *Acinetobacter baumannii* is largely attributed to its ability to persist and resist. The niches encountered during infection vary significantly from the more commonly studied laboratory setting, and consequently, the genes responsible for *in vivo* pathogenesis have yet to be fully elucidated. This study utilized the *A. baumannii* AB5075-UW transposon mutant library with unbiased genome-wide transposon sequencing to identify the genetic basis for survival and fitness during pneumonia and septicaemia infections. We identified 128 genes essential for in-host survival, including 22 required for survival in all tissues. Additionally, 302 genes with significantly altered fitness *in vivo* were also identified. Tissue specificity was observed, highlighting the importance of genes associated with aa biosynthesis in the lungs, cell shape and structure in the kidneys and metal acquisition during septicaemia. The majority (89%) of the genes with aberrant fitness were constituents of the core *A. baumannii* genome. The findings were validated using a subset of targeted mutants, including those required for infection (*phoB*, *cysI* and *hom*) or specifically septicaemia (*corA*, *lepA* and *purN*) or pneumonia (*argC*, *hisC* and *leuD*), confirming that these observations were a result of specific *in vivo* fitness defects rather than aberrant *in vitro* growth. Taken together, these data provide the first global profile of genes required for *in vivo* fitness of *A. baumannii* during different disease states and growth in different tissues.

**Impact Statement**

*Acinetobacter baumannii* is an important human pathogen responsible for a range of life-threatening infections. The rapid global emergence of multi-, extensively- and pandrug-resistant strains has seen morbidity and mortality rates rise, with untreatable infections now a reality. To address the current void of new therapeutics in development, a more complete understanding of *A. baumannii in vivo* survival is required. Here, we used unbiased genome-wide transposon insertion sequencing to identify genes required for survival and bacterial fitness *in vivo* and examined tissue and infection route specificity. We identified 128 and 302 genes that are essential for survival and influence *in vivo* fitness, respectively, and confirmed that 89% of these were part of the *A. baumannii* core genome, suggesting that these are species-wide virulence determinants. Advancing our understanding of this important human pathogen and the mechanisms responsible for disease will greatly assist in the development of new therapeutic or preventative approaches.

## Data Summary

Raw reads for the *Acinetobacter baumannii* transposon insertion sequencing are provided in the European Nucleotide Archive under study PRJEB83056. Accessions for individual samples are given in Table S3 (available in the online Supplementary Material).

## Introduction

*Acinetobacter baumannii* is a causative agent of bacteraemia, endocarditis, pneumonia and meningitis [1,2]. It is responsible for >10% of all nosocomial infections, and its inherent resistance to disinfectants and desiccation, combined with genomic plasticity, has led to the rapid emergence of multi-, extensively- and pandrug-resistant isolates [3,4]. Multidrug-resistant strains are now responsible for >60% of all ventilator-associated pneumonia cases (mortality rate >35 %), and it is estimated that 50% of all *A. baumannii* infections in the USA are caused by carbapenem-resistant strains [5,6]. Accordingly, the World Health Organization has classified carbapenem-resistant *A. baumannii* as the number one ‘critical’ pathogen urgently requiring new therapeutics [7]. However, with only a limited number of traditional virulence factors, the mechanisms by which *A. baumannii* causes disease are still poorly understood, and many genes critical to *in vivo* survival and fitness remain to be identified [8].

Recent improvements in high-throughput sequencing technology have seen an explosion in bacterial functional genomics, expanding genotype–phenotype relationships. A number of similar techniques, utilizing saturation transposon mutagenesis combined with high-throughput sequencing of insertion sites, can generate high-resolution, genome-scale information on gene essentiality and fitness [9,11]. These approaches have been applied to Gram-positive and Gram-negative pathogens under a range of *in vitro* and *in vivo* conditions. Studies in *Escherichia coli*, *Salmonella enterica*, *Pseudomonas aeruginosa*, *Staphylococcus aureus* and *Streptococcus pneumoniae* have expanded our understanding of the factors associated with disease [12,16].

A limited number of studies have applied transposon insertion sequencing to *A. baumannii* infections, utilizing different *in vitro* (human serum) and *in vivo* disease models (*Galleria mellonella* and murine) and different strains (ATCC17978 and AB5075-UW) [17,20]. Collectively, these studies have identified numerous genes and cellular pathways essential for survival and have confirmed the importance of nutrient acquisition, stress response systems, capsule and lipooligosaccharide biosynthesis for survival *in vivo* [17,19,21]. However, due to limitations stemming from infection bottlenecks, low mutant library diversity or the *A. baumannii* strain used, none of these studies provide a comprehensive view of the genes contributing to a mammalian infection [18,20,22]. Furthermore, the importance of specific *A. baumannii* genes to fitness in different tissues and disease states has not been explored.

This study aimed to identify *A. baumannii* genes important for survival and fitness during different disease states and growth in different tissues. Utilizing the near-saturated AB5075-UW transposon mutant pool [22], we performed unbiased transposon sequencing following *in vivo* exposure to identify genes essential for survival and fitness during acute murine pneumonia and septicaemia infections. Bioinformatic analyses demonstrated that the majority of these genes were part of the core genome. We validated a selection of these (*argC*, *corA*, *cysI*, *hisC*, *ho*m, *lepA*, *leuD*, *phoB* and *purN*) using single-gene mutants and *in vivo* competitive infections, confirming that these were specifically required for *in vivo*, but not *in vitro* fitness. These data provide a comprehensive assessment of the genetic basis of *A. baumannii* pathogenesis during different infections and tissue-specific colonization, highlighting numerous potential targets for therapeutic interventions.

## Methods

### Bacterial strains and culture conditions

Bacterial strains used in this study are described in Table S1. Unless otherwise indicated, bacteria were cultured in Luria-Bertani (LB) with shaking at 37 °C. Media were supplemented with 1.5% (w/v) agar bacteriological no. 1 (Oxoid) and 5 µg ml^−1^ tetracycline hydrochloride (Sigma), as required.

The *A. baumannii* AB5075-UW transposon pool infectious doses were prepared immediately following a 1-h outgrowth in LB broth at 37 °C with aeration. Briefly, the outgrown pools were pelleted at 8,000 ***g*** for 10 min, washed with PBS and adjusted to a density of 5×10^7^ and 2×10^9^ c.f.u. ml^−1^, for intraperitoneal and intranasal infection, respectively. The intraperitoneal doses were mixed at a 1 : 1 ratio with 12% w/v porcine mucin (Sigma) to achieve the final dose of 5×10^6^ c.f.u./200 µl in 6% mucin as described below.

### Murine infection models

*In vivo* experiments were performed with ethical approval under project reference E/1689/2016 /M, using 6–8-week-old female immunocompetent BALB/c mice, housed at the Monash Animal Research Facility, Monash University. Animals were infected with 1×10^8^ c.f.u. of *A. baumannii* via the intranasal route (pneumonia) immediately following anaesthesia with vaporized isoflurane, or either 5×10^6^ (for the transposon mutant pool) or 5×10^4^ c.f.u. of *A. baumannii* (for validation) in 6% w/v porcine mucin (Sigma) via intraperitoneal injection (septicaemia) in accordance with previously described methods [23,24]. Animals (four or five per replicate) were euthanized 8 h post-infection, and the lungs, blood, kidneys, liver and spleen were collected.

### Sample preparation and DNA extraction

Lungs (pneumonia), kidneys, liver and spleen (septicaemia) were harvested and homogenized in PBS. Blood (septicaemia) was collected by cardiac puncture and immediately mixed with an equivalent volume of PBS. A sample from each was taken for bacterial enumeration. The remaining volume was plated across 10 LB agar plates (without selection) and incubated for 3 h at 37 °C, after which bacteria were recovered by washing twice with PBS. Bacterial suspensions were pelleted by centrifugation at 8,000 ***g*** for 10 min and washed as above. Bacterial DNA was extracted using the QIAGEN DNeasy blood and tissue extraction kit, in accordance with manufacturer’s instructions, with minor modifications. Briefly, each sample was divided in two and proteinase K digestion was performed over 14 h. The infectious dose (input pool) was prepared in accordance with the tissue samples as described above. Briefly, either 2.5×10^6^ c.f.u. (intraperitoneal) or 1×10^8^ c.f.u. (intranasal) of the inoculum was plated onto either LB agar or inoculated into LB broth, incubated at 37 °C for 3 h, rinsed from the agar plates by washing twice with PBS, pelleted at 8,000 ***g*** for 10 min and washed as above, prior to DNA extraction. DNA concentrations were confirmed using the Qubit DNA high sensitivity kit (ThermoFisher).

### Library preparation and sequencing

Illumina compatible libraries were prepared for each sample individually using the terminal deoxynucleotidyl transferase method described previously [22]. DNA was purified using 1.5 volumes of AxyPrep Magnetic (MAG) PCR Clean-Up Beads (Axygen) for the retention of fragments between 100 and 1,000 bp and average fragment size determined at appropriate steps using a Fragment analyser (Agilent). Primers used in this study are described in Table S2. The amount of clusterable DNA in each library was quantified using KAPA Illumina library quantification kit (Millennium Science). Illumina libraries generated for each sample were pooled, combining equal molar concentrations of DNA from each organ and animal and sequenced on an Illumina MiSeq at the Ramaciotti Centre for Genomics (University of New South Wales) using custom sequencing primer AP1823 with sequencing protocols as described previously [25].

### Data analysis

Sequencing reads were trimmed, mapped to the AB5075-UW chromosome (NZ_CP008706) and assigned to genomic features as follows: the transposon tag (‘TAAGAGACAG’) was supplied as a secondary index sequence and only those reads containing a transposon sequence were retained. Examination of the reads with FastQC [26] showed a C-nucleotide skew towards the 3′ end of the read, with roughly 20 % of reads containing five or more 3′ C-nucleotides derived from the C-terminal tails introduced during library construction. CutAdapt was used to trim 3′ C-strings of five or more, and the processed reads were analysed and mapped to the AB5075-UW genome and transposon insertion plot files generated using the bacteria-tradis script within the Bio-TraDIS toolkit [25,27]. Sequencing depth was assessed using the seq_saturation_test.py script (https://github.com/francesca-short/tradis_scripts). The solid (plate) and liquid outgrown input doses from replicate two were found to be under-sequenced; therefore, both samples were combined to provide sufficient coverage of the library diversity. Insertion sites were assigned to genomic features using the tradis_insert_sites_py3.py script (https://github.com/francesca-short/tradis_scripts). Insertion sites that mapped to the last 10% of the 3′ end were discarded in order to avoid measuring effects from non-inactivating mutations. Gene-wise read abundance was compared between input and output samples using edgeR/limma as implemented in Degust, with hits defined as a log_2_ fold change of < −1 or >1, with a false discovery rate (FDR) <0.01 [28]. Gene essentiality was determined using the tradis_essentiality function of the Bio-TraDIS toolkit. Protein coding sequences were assigned to functional categories (Gene Ontology and Clusters of Orthologous Groups) using EggNOG Mapper [29,30].

### Pangenome analysis

An *A. baumannii* pangenome was generated from 172 complete genomes available in the NCBI as of 20 March 2020 using Panaroo version 1.1.2 [31]. For consistency of annotation, genome sequences were first annotated using Prokka version 1.14.6 [32], with preferential assignment of genes based on the AB5075-UW annotation (set using the --proteins flag). Panaroo version 1.1.2 was then run using default parameters. These parameters include initial cluster thresholds of 98% amino acid identity/98% length, a subsequent threshold for defining gene families of 70% sequence identity, splitting of paralogues into separate clusters and a gene refinding step. Based on the occurrence across the 172 genomes, the AB5075-UW coding sequences were defined as core (present in >98% of genomes), soft-core (95–98%), shell (15–95%) or cloud (<15%).

### Genetic validation of single mutants

Single transposon mutants were obtained from the *A. baumannii* ordered library [22] and validated by PCR using Taq DNA polymerase (Thermo Scientific) and gene-specific oligonucleotides (Table S2). Southern blot hybridization confirmed the presence of a single transposon insertion. Briefly, transposon insertions were detected using a DIG-labelled tetracycline-specific probe generated using the DIG labelling kit (Roche) and primers AP1561 and AP1562. Genomic DNA was digested using combinations of *Bgl*II, *Mfe*I, *Pvu*I, *Pvu*II, *PflM*I, *Sca*I and/or *Spe*I (NEB) in accordance with the manufacturer’s instructions. Equal concentrations of digested DNA were separated by gel electrophoresis using 1.2% w/v agarose and transferred to a nitrocellulose membrane, and the membrane was probed with anti-digoxigenin antibody (Roche) and visualized by chemiluminescence using CDP star (Roche), in accordance with the manufacturer’s instructions. The signals were detected using the ChemiDoc MP Imaging system (Bio-Rad) under signal accumulation mode.

### Validation of single mutant phenotypes

Single mutants were evaluated for *in vitro* growth kinetics under standard conditions (LB at 37 °C with aeration), with capsule isolation and Alcian blue staining performed as described previously [33].

### Competitive fitness *in vitro* and *in vivo*

Bacterial competitive fitness was performed *in vitro* and *in vivo*. Briefly, bacterial densities from 1 : 1 mixed cultures or infections were enumerated 8 h post-inoculation/infection on LB agar with (selection for transposon mutant only) and without tetracycline (selection for both strains). The WT bacterial burdens were calculated by subtracting the number of mutant c.f.u. from that of the total. Competitive indices were calculated by dividing the recovered mutant c.f.u. ml^−1^ by that of the WT at the end of the experiment, by the same ratio calculated from the starting inoculum. Statistically significant differences between recovered c.f.u. ml^−1^ values were determined using a Mann–Whitney t-test, with a *P* value <0.05 considered significant.

## Results and discussion

### Determining the genes required for *A. baumannii* infection

**Fig. 1.**
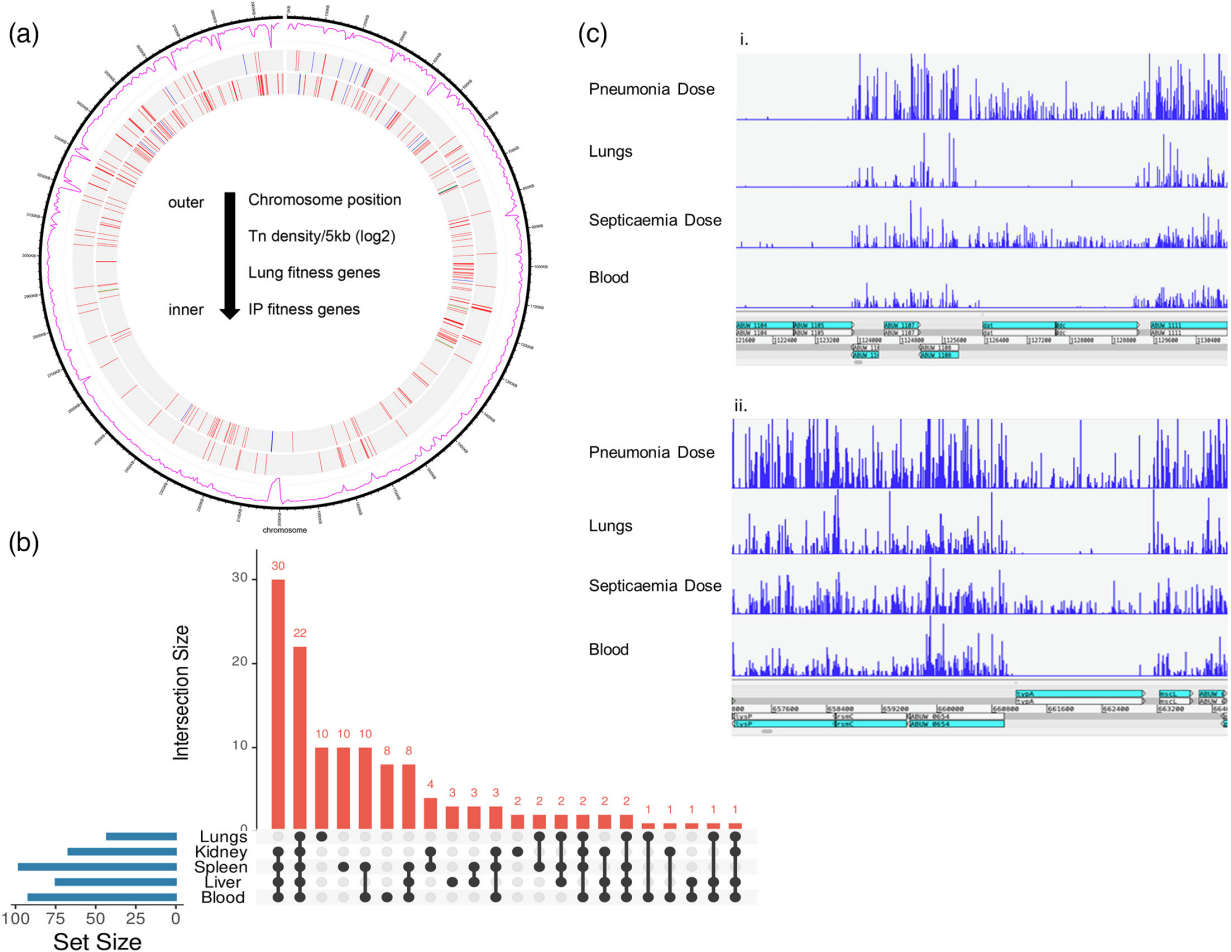
Distribution of transposon insertions and genes essential for survival *in vivo*. (**a**) Overview of transposon insertion data showing chromosome location, insertion density per 5 kb (log_2_) and genes identified as required for fitness during infection by either the intranasal or intraperitoneal route (combined fitness genes identified from blood, kidneys, liver and spleen). Fitness genes are coloured red for decreased fitness, blue for increased fitness and green if fitness was decreased in one tissue but increased in another. Note that bar size is standard and not proportional to gene size. The plot was generated using ShinyCircos [110]. (**b**) The distribution of the 128 genes found to be essential for survival. Individual gene locus tags represented in the UpSet plot (generated using https://asntech.shinyapps.io/intervene/) are provided in Table S5. (**c**) Insertion plots for the pneumonia and septicaemia doses aligned with the lungs and blood samples, respectively, highlighting *dat/ddc* (i) and *typA* (ii) mutants were conditionally essential irrespective of infection route.

To assess the genes required for *in vivo* survival and fitness of *A. baumannii*, we first sought to maximize the inclusion of mutants from our Tn-Seq library pool. We determined that an inoculum of 5×10^6^ c.f.u. (septicaemia) and 1×10^8^ c.f.u. (pneumonia), containing ~11 and >200 copies of each unique transposon insertion (assuming a library density of 450,000 as previously reported) [22], respectively, reproducibly caused disease. Mice were infected via intraperitoneal injection or intranasal inoculation, and blood, kidneys, liver and spleen (septicaemia model) or lungs (pneumonia model) were collected 8 h post-infection (Fig. S1). The bacterial load in each tissue was consistent between mice (Fig. S2), and bacterial genomic DNA was extracted from each tissue individually following a 3 h outgrowth and used to generate TraDIS libraries that were sequenced using Illumina MiSeq. Between 73 and 80% of the reads were mapped uniquely to AB5075-UW (Fig. 1a), and analysis of the transposon insertion density revealed ~180,000 unique insertion sites. Minimal loss of library diversity was observed between the input and output pools, and comparisons between biological replicates showed excellent correlation (*R*^2^ ≥0.9 reads per gene for all samples, Fig. S3). Sequencing statistics and all data are provided in Tables S3 and S4, respectively.

### Genes essential for survival *in vivo*

To accurately determine the genes required for pathogenesis, we first identified those conditionally essential *in vivo* (Table 1) using the ‘tradis_essentiality’ pipeline [25], comparing the bimodal distribution of insertion indices normalized to gene length. This identified 128 genes essential in at least 1 tissue. Of these, 22 genes were essential in all tissues; however, 14 had reduced representation in the input pool. The analysis identified genes required for specific diseases, including 10 and 30 genes for pneumonia and septicaemia, respectively, in addition to those required for survival in specific areas (eight genes in the blood, two in the kidney, three in the liver and 10 in the spleen, Fig. 1b, c and Table S5).

**Table 1.** Genes essential for survival in at least one tissue

Locus tag	Gene name	Function	Essential*					Literature comparison‡				
			Spleen	Lung	Liver	Kidney	Input†	Gallagher	Gebhardt	Wang§	Wang¶	Locus tag**
ABUW_0097	*serB*	Phosphoserine phosphatase	1	1	1	1	0	0	0	0	1	A1S_3386
ABUW_0331	*serA*	d-3-Phosphoglycerate dehydrogenase	1	1	1	1	0	0	0	0	1	A1S_3152
ABUW_0903	ABUW_0903	tRNA-Leu	1	1	1	1	0	0	0	0		na
ABUW_1109	*dat*	Diaminobutyrate-2-oxoglutarate aminotransferase	1	1	1	1	0	0	0	0	0	A1S_2454
ABUW_2192	ABUW_2192	ABC-1 domain protein	1	1	1	1	0	0	0	0	0	A1S_1644
ABUW_2515	ABUW_2515	Hypothetical protein	1	1	1	1	0	1	0			na
ABUW_3821	ABUW_3821	Spore coat polysaccharide biosynthesis protein spsC	1	1	1	1	0	0	1			na
ABUW_3832	*ptp*	Protein-tyrosine-phosphatase ptp	1	1	1	1	0	0	1	0	0	A1S_0050
ABUW_0460	ABUW_0460	Hypothetical protein	1	1	1	1	0.5	0	1	0	0	A1S_3033
ABUW_0655	*typA*	GTP-binding protein TypA/BipA	1	1	1	1	0.5	0	1	0	0	A1S_2835
ABUW_1110	*ddc*	Diaminobutyrate decarboxylase	1	1	1	1	0.5	0	0	0	0	A1S_2453
ABUW_1447	ABUW_1447	Hypothetical protein	1	1	1	1	0.5	0	1	0	0	A1S_2250
ABUW_2136	*aroC*	Chorismate synthase	1	1	1	1	0.5	1	0	0	0	A1S_1694
ABUW_3276	*trx*	Thioredoxin	1	1	1	1	0.5	0	0	0	0	A1S_0606
ABUW_3298	*pcnB*	Poly(A) polymerase	1	1	1	1	0.5	0	0	0	1	A1S_0583
ABUW_3306	ABUW_3306	Multi-sensor hybrid histidine kinase	1	1	1	1	0.5	0	0	0	1	A1S_0574
ABUW_3368	*engC*	GTPase	1	1	1	1	0.5	0	0	0	0	A1S_0531
ABUW_3652	*ribD*	Riboflavin biosynthesis protein RibD	1	1	1	1	0.5	1	0	1		A1S_0221
ABUW_3731	*atpC*	ATP synthase F1, epsilon subunit	1	1	1	1	0.5	0	0	0	0	A1S_0156
ABUW_3765	ABUW_3765	tRNA-Gly	1	1	1	1	0.5	0	0			na
ABUW_3823	ABUW_3823	Putative UDP-galactose phosphate transferase (WeeH)	1	1	1	1	0.5	0	1			na
ABUW_3828	ABUW_3828	Hypothetical protein	1	1	1	1	0.5	0	1			na
ABUW_0262	ABUW_0262	ABC transporter ATP-binding protein	1	1	1	0	0	0	0	0	1	A1S_3221
ABUW_0332	ABUW_0332	FAD-linked oxidase domain protein	1	1	1	0	0	0	0	0	1	A1S_3151
ABUW_0532	*yajC*	Preprotein translocase, YajC subunit	1	1	0	1	0	0	0	1		A1S_2913
ABUW_0722	*cysH*	Phosphoadenosine phosphosulphate reductase	0	1	1	1	0	0	1	1		A1S_2775
ABUW_0850	*lolA*	Outer membrane lipoprotein carrier protein LolA	1	1	0	1	0.5	1	0	1		A1S_2729
ABUW_0957	*xerC*	Tyrosine recombinase XerC	1	1	1	0	0	0	0	1		A1S_2629
ABUW_0980	*purN*	Phosphoribosylglycinamide formyltransferase	0	1	1	0	0.5	0	0	0	1	A1S_2606
ABUW_1012	ABUW_1012	ExsB protein	1	1	1	0	0.5	0	0	0	0	A1S_2542
ABUW_1029	*lepA*	GTP-binding protein LepA	0	1	0	0	0	0	0	1		A1S_2522
ABUW_1032	*rnc*	Ribonuclease III	1	1	0	0	0.5	0	0	1		A1S_2519
ABUW_1204	*trpG*	Para-aminobenzoate/anthranilate synthase glutamine amidotransferase component	1	1	0	0	0.5	0	0	0	1	A1S_2355
ABUW_1224	*ahcY*	Adenosylhomocysteinase	0	1	0	0	0	0	0	0	0	A1S_2334
ABUW_1228	*lipA1*	Lipoic acid synthetase	0	1	0	0	0	0	1	0	0	A1S_2329
ABUW_1246	*queF*	NADPH-dependent 7-cyano-7-deazaguanine reductase (EsvE1)	0	1	0	0	0	0	0	0	0	A1S_2313
ABUW_2198	*hup*	DNA-binding protein HU	0	1	0	0	0	0	0	0	0	A1S_1637
ABUW_2208	ABUW_2208	Adenylate/guanylate cyclase	0	1	0	0	0	0	1	0	0	A1S_1625
ABUW_2248	ABUW_2248	6-Pyruvoyl tetrahydrobiopterin synthase	0	1	0	0	0	0	0	0	0	A1S_1566
ABUW_2342	*ychF*	GTP-binding protein YchF	0	1	0	0	0	0	1	0	0	A1S_1481
ABUW_2431	*umuD*	DNA polymerase V component	0	1	0	0	0	0	0	0	0	A1S_1389
ABUW_2741	*vfr*	Cyclic nucleotide-binding domain protein	0	1	0	0	0	0	1	0	0	A1S_1182
ABUW_3260	ABUW_3260	Putative two-component response regulator	0	1	0	0	0.5	0	1	0	0	A1S_0621
ABUW_3393	*eda*	khg/kdpg aldolase	1	0	1	1	0	0	1	0	0	A1S_0484
ABUW_3448	ABUW_3448	Glycosyl transferase, group 1	1	0	1	1	0	1	0	0	1	A1S_0430
ABUW_3522	*recB*	Exonuclease V beta chain	1	0	1	1	0	0	0	1		A1S_0354
ABUW_3609	ABUW_3609	DNA-binding protein H-NS	1	0	1	1	0	0	0	1		A1S_0357
ABUW_3642	ABUW_3642	Putative periplasmic carboxyl-terminal protease	1	0	1	1	0	0	0	0	0	A1S_0231
ABUW_3830	ABUW_3830	UDP-glucose/GDP-mannose dehydrogenase	1	0	1	1	0	0	0	0	0	A1S_0052
ABUW_0093	*nusB*	Transcription antitermination factor NusB	1	0	1	1	0	1	0	0	0	A1S_3390
ABUW_1526	ABUW_1526	Hypothetical protein	1	0	1	1	0	1	0	0	0	A1S_2195
ABUW_1608	*miaA*	tRNA delta [2]-isopentenylpyrophosphate transferase	1	0	1	1	0	0	1	0	0	A1S_2113
ABUW_2209	ABUW_2209	Histidine triad protein	1	0	1	1	0	0	0	1		A1S_3233
ABUW_3083	*smpB*	SsrA-binding protein	1	0	1	1	0	0	0	0	0	A1S_0844
ABUW_3231	*rpe*	Ribulose-phosphate 3-epimerase	1	0	1	1	0	0	0	0	0	A1S_0705
ABUW_3885	*purK*	Phosphoribosylaminoimidazole carboxylase, ATPase subunit	1	0	1	1	0	0	1	0	1	A1S_2963
ABUW_3902	*trmE*	tRNA modification GTPase TrmE	1	0	1	1	0	0	1	0	0	A1S_2979
ABUW_0483	ABUW_0483	Hypothetical protein	1	0	1	1	0	0	0			na
ABUW_0695	ABUW_0695	tRNA-Asp	1	0	1	1	0	0	0			na
ABUW_1013	ABUW_1013	Radical SAM domain protein	1	0	1	1	0	0	0	0	0	A1S_2540
ABUW_1174	*bauC*	Ferric acinetobactin transport system permease	1	0	1	1	0	0	1	1		A1S_0052
ABUW_1177	*bauA*	Ferric acinetobactin receptor	1	0	1	1	0	1	0	1		A1S_0705
ABUW_1614	*ppiB*	Peptidyl-prolyl cis-trans isomerase, cyclophilin type	1	0	1	1	0	0	0	1		A1S_2109
ABUW_1628	ABUW_1628	Hypothetical protein	1	0	1	1	0	0	0	1		A1S_0231
ABUW_2719	*ahpC*	Peroxiredoxin	1	0	1	1	0	0	0	0	0	A1S_1205
ABUW_3048	*minC*	Septum site-determining protein MinC	1	0	1	1	0.5	0	0	1		A1S_2113
ABUW_3523	*recC*	Exonuclease V gamma chain	1	0	1	1	0.5	0	1	0	0	A1S_0354
ABUW_3638	*pbpG*	d-Alanyl-d-alanine carboxypeptidase family protein	1	0	1	1	0.5	0	0	1		A1S_3390
ABUW_0156	*aceF*	Pyruvate dehydrogenase complex dihydrolipoamide acetyltransferase	1	0	1	1	0.5	0	0	0	0	A1S_3327
ABUW_0251	ABUW_0251	Hypothetical protein	1	0	1	1	0.5	0	1	0	0	A1S_3233
ABUW_1252	ABUW_1252	Hypothetical protein	1	0	1	1	0.5	0	0			na
ABUW_1267	ABUW_1267	Hypothetical protein	1	0	1	1	0.5	0	0			na
ABUW_2821	ABUW_2821	tRNA-Ser	1	0	1	1	0.5	0	0			na
ABUW_1740	*rseP*	Membrane-associated Zn-dependent protease	0	0	1	1	0	0	1	0	0	A1S_1970
ABUW_0164	*pdxH*	Pyridoxamine 5′-phosphate oxidase	0	0	1	1	0	1	0	1		A1S_3319
ABUW_0381	ABUW_0381	DEAD/DEAH box helicase	1	0	1	0	0	0	0	0	0	A1S_3104
ABUW_0388	*corA*	Magnesium and cobalt transport protein	1	0	0	1	0	0	0	1		A1S_3098
ABUW_0719	*cafA*	Ribonuclease G	1	0	0	1	0	0	1	0	1	A1S_2777
ABUW_1176	*bauB*	Ferric acinetobactin transport system periplasmic binding protein	1	0	0	1	0	0	1	0	0	A1S_2386
ABUW_1208	*ubiC*	Chorismate lyase	1	0	1	0	0	0	0	0	0	A1S_2350
ABUW_1612	ABUW_1612	Hypothetical protein	1	0	1	0	0	0	0			na
ABUW_2276	ABUW_2276	Transcriptional regulator, ArsR family	1	0	1	0	0	0	0	0	0	A1S_1539
ABUW_2290	ABUW_2290	Hypothetical protein	1	0	1	0	0	0	0	0	0	A1S_1526
ABUW_2304	*ruvC*	Crossover junction endodeoxyribonuclease RuvC	1	0	1	0	0.5	0	1	1		A1S_1514
ABUW_2357	ABUW_2357	Aspartate aminotransferase	1	0	1	0	0.5	0	0	0	1	A1S_1468
ABUW_2786	ABUW_2786	Transcriptional regulator, GntR family	1	0	1	0	0.5	0	0	0	0	A1S_1103
ABUW_2362	*cysE*	Serine acetyltransferase	1	0	0	0	0	0	1	0	0	A1S_1463
ABUW_2673	ABUW_2673	Hypothetical protein	1	0	0	0	0	1	0			na
ABUW_3261	ABUW_3261	Putative anti-anti-sigma factor	0	0	1	0	0	0	0	1		A1S_0620
ABUW_3305	*cysM*	Cysteine synthase B	1	0	0	0	0	0	0	0	0	A1S_0575
ABUW_0537	ABUW_0537	–	1	0	0	0	0	0	0			na
ABUW_0644	ABUW_0644	Hypothetical protein	1	0	1	0	0	0	0	0	0	A1S_2845
ABUW_2589	ABUW_2589	Transcriptional regulator, DUF24 family	1	0	1	0	0	0	0	0	0	A1S_1280
ABUW_2704	ABUW_2704	Hypothetical protein	1	0	0	0	0	1	0			na
ABUW_3540	*rbfA*	Ribosome-binding factor A	1	0	0	0	0	0	0	0	0	A1S_0338
ABUW_0277	ABUW_0277	tRNA-Ala	0	0	0	1	0	0	0			na
ABUW_0744	ABUW_0744	Hypothetical protein	1	0	0	1	0	0	0			na
ABUW_0863	ABUW_0863	Hypothetical protein	1	0	0	1	0	0	0	0	0	A1S_3830
ABUW_1072	*idh*	Isocitrate dehydrogenase, NADP-dependent	1	0	0	0	0.5	0	0	0	0	A1S_2477
ABUW_1172	ABUW_1172	Hypothetical protein	1	0	0	0	0.5	0	0			na
ABUW_1173	*bauD*	Ferric acinetobactin transport system permease	1	0	0	0	0.5	0	1	0	1	A1S_2389
ABUW_1200	*trpC*	Indole-3-glycerol phosphate synthase	1	0	0	0	0.5	0	0	0	1	A1S_2360
ABUW_1221	ABUW_1221	Sensory box protein	1	0	0	1	0.5	0	0	0	0	A1S_2337
ABUW_1279	ABUW_1279	Hypothetical protein	1	0	0	1	0.5	0	0			na
ABUW_1516	ABUW_1516	Hypothetical protein	1	0	1	0	0.5	1	1			na
ABUW_1663	ABUW_1663	Hypothetical protein	0	0	0	0	0	0	0	0	0	A1S_3782
ABUW_1734	ABUW_1734	Hypothetical protein	1	0	0	0	0	0	1	1		A1S_1976
ABUW_1752	ABUW_1752	Hypothetical protein	0	0	0	0	0	0	0	0	0	A1S_3748
ABUW_1811	*argC*	*N*-Acetyl-gamma-glutamyl-phosphate reductase	1	0	0	0	0	0	0	0	0	A1S_1913
ABUW_1814	*clpA*	ATP-dependent Clp protease ATP-binding subunit	0	0	1	0	0	0	0	0	0	A1S_1910
ABUW_2262	*spoOJ*	Chromosome partitioning protein ParB	0	0	0	0	0	0	1	0	0	A1S_1552
ABUW_2686	ABUW_2686	Hypothetical protein	0	0	0	0	0	0	0	0	0	A1S_3632
ABUW_2689	ABUW_2689	Hypothetical protein	0	0	0	1	0	0	0	0	0	A1S_3631
ABUW_2746	*hisZ*	ATP phosphoribosyltransferase regulatory subunit	1	0	0	0	0	0	0	1		A1S_1178
ABUW_2813	ABUW_2813	Hypothetical protein	0	0	1	0	0	0	0			na
ABUW_2919	ABUW_2919	Hypothetical protein	1	0	0	0	0	1	0	0	0	A1S_3586
ABUW_3192	ABUW_3192	Hypothetical protein	0	0	0	0	0	0	1			na
ABUW_3246	*pabB*	P-aminobenzoate synthetase PabB	0	0	1	0	0	0	0	0	0	A1S_0689
ABUW_3247	*hisC*	Histidinol-phosphate aminotransferase	1	0	0	0	0	0	0	0	1	A1S_0688
ABUW_3466	ABUW_3466	HAD-superfamily subfamily IB hydrolase	1	0	0	0	0	0	0	0	1	A1S_0415
ABUW_3627	*dksA*	DnaK suppressor protein	1	0	0	0	0	0	0	0	1	A1S_0248
ABUW_3636	*hom*	Homoserine dehydrogenase	0	0	0	0	0	0	0	0	1	A1S_0239
ABUW_3829	ABUW_3829	Hypothetical protein	0	0	0	0	0	0	0			na
ABUW_3846	*dsbA*	Thiol:disulphide interchange protein DsbA	1	0	0	0	0	0	1	0	0	A1S_0037
ABUW_2303	ABUW_2303	Hypothetical protein	0	0	0	1	0	0	0			na
ABUW_0800	ABUW_0800	Hypothetical protein	0	0	0	0	0.5	0	0			na
ABUW_2227	ABUW_2227	Hypothetical protein	1	0	0	0	0.5	1	0			na
ABUW_2388	ABUW_2388	Hypothetical protein	1	0	0	0	0.5	0	0			na

*Essentiality determined relative to the input concentration, whereby a value of zero is non-essential, 0.5 refers to reduced frequency and one is essential.

†Input pools outgrown for DNA extraction using two separate methods (on plates, as per tissues or in liquid broth for the equivalent period of time), to determine the impact of different outgrowth conditions on the insertion indices recovered. The cumulative score from both conditions was used for determination of essentiality.

‡Comparison of genes identified as essential by Gallagher *et al*. [22], Gebhardt *et al*. [17] and Wang *et al*. (in rich media only) [20]. A value of zero is non-essential and one is essential, and blank values refer to instances where no homologue was identifiable.

§Comparison of genes identified as essential for growth in rich media by Wang *et al*. [20]. A value of zero is non-essential and one is essential, and blank values refer to instances where no homologue was identifiable.

¶Comparison of genes identified as essential for persistence in the murine lung by Wang *et al.* [20]. A value of zero is non-essential, one is essential and blank values refer to instances where either no homologue was identifiable or the gene was previously identified as essential for growth in rich media during the same experiment.

**Locus tag for ATCC17978. na refers to no homologue.

Functional grouping of the 128 essential genes found that hypothetical proteins were the most prevalent (*n*=33), followed by nucleotide biosynthesis and RNA (*n*=22). Similarly, six genes were associated with DNA binding and repair, in addition to nine transcriptional regulators. Notably, genes associated with iron scavenging (*bauABCD*), amino acid and micronutrient biosynthesis and metabolism (including amine and polysaccharide biosynthesis and cysteine/sulphur metabolism) also featured prominently (*n*=28).

Excluding those with reduced representation in the input pool, the eight remaining genes identified as essential in all tissues were attributed to diverse functional groups, including a tRNA, l-serine, amine and polysaccharide biosynthesis, an ABC transporter, a hypothetical protein and a cell envelope-associated tyrosine phosphatase. While the conditional essentiality of the l-serine biosynthetic components, *serAB*, may reflect its diversity as a precursor for the synthesis of other molecules, in *Mycobacterium tuberculosis*, this pathway is essential and its disruption in *Burkholderia gladioli*, *Burkholderia pseudomallei* and *Brucella abortus* is known to impair virulence [34,36]. However, in *Oreochromis niloticus* (fish) infected with *Edwardsiella tarda*, the expression of host serine metabolism genes has been shown to reduce reactive oxygen species by enhancing glutathione synthesis [37]. It is possible that l-serine production may be important for the modulation of the host immune responses rather than bacterial homeostasis. However, this hypothesis requires further validation, given *serC* (a vital component in the l-serine biosynthetic pathway) was not identified in this study.

Of the 10 genes essential for survival in the lungs, 9 were associated with RNA and DNA, while 10 genes (of 30) essential for survival in all systemic tissues were also in this category. The remaining categories consisted of hypothetical proteins (*n*=6), iron and micronutrient biosynthesis (*n*=5) and other cellular functions (including division, periplasmic proteins and other enzymes, *n*=21). Interestingly, the virulence factor regulator, Vfr*,* was conditionally essential in the lungs. In *P. aeruginosa*, Vfr functions via cAMP-dependent and independent mechanisms regulating a range of virulence genes, including the type III secretion system, type IV pili, exotoxin A and quorum sensing [38,39]. In *A. baumannii*, the expression of *vfr* is known to be upregulated in response to high cAMP, such as in a *cpdA* mutant background (which accumulates intracellular cAMP), and is dependent on the adenylate cyclase, CavA (ABUW_2208), driving biofilm formation and modulation of c-di-GMP levels, in response to cAMP [39,41]. Interestingly, while *cpdA* was found to be essential in the spleen and kidney, *cavA* was conditionally essential in all tissues except the lungs, highlighting the interconnected pathways regulating virulence. While the transcriptional impact of *cpdA* and *cavA* has been explored previously in *A. baumannii* [39,41], these observations highlight the need for detailed characterization of Vfr, to decipher its regulon and identify putative effector protein(s) that function in concert to modulate cellular responses to secondary messengers [41].

Of the 128 genes identified during this study, 15 and 22 have previously been identified as essential in AB5075-UW and ATCC17978, respectively, under laboratory conditions [20,22]. Of these, six had reduced representation in our input pool, though only four are consistent between the two previously published datasets (Table 1). Comparison of those genes identified as essential *in vivo* during this study with those required for persistence in the murine lung or survival in *Galleria* identified 18 and 31 genes in agreement, respectively [17,20]. However, only eight of the genes essential for persistence in the lung were essential in this tissue in our data set, and only three genes were consistent between all datasets. These comparisons highlight the impact different models and strains make to *A. baumannii* pathogenesis [42].

### Genes required for *in vivo* fitness in all tissues

In addition to conditionally essential genes, we sought to identify those genes that contribute to *in vivo* fitness using a log_2_ fold change < −1 and a FDR <0.01. This analysis identified 281 genes with reduced *in vivo* fitness (Tables 2 and S6), including 42 previously identified as conditionally essential and 7 that were significantly attenuated in 1 tissue while showing increased fitness in another (*aceE*, *dacC*, *mreB*, *mreC*, *mrdA*, *mrdB* and ABUW_0523). This analysis identified 18 genes attenuated in all tissues irrespective of infection route, including 4 conditionally essential in all tissues (*dat*, *ddc*, *typA* and ABUW_3821) and 2 following septicaemia infection (ABUW_3448 and ABUW_3830), respectively.

**Table 2.** Genes important for bacterial fitness and/or essential for survival during septicaemia and/or pneumonia infection

Tissue specificity	Locus tag	Gene name	Known or predicted function	Transposon insertion rate fold-change (log_2_)					Essential*
				Blood	Liver	Spleen	Kidney	Lung	
All tissues (18 genes)	ABUW_1109	*dat*	Diaminobutyrate-2-oxoglutarate aminotransferase	−10.45	−6.23	−5.70	−6.11	−3.98	1
	ABUW_0655	*typA*	GTP-binding protein TypA/BipA	−7.60	−8.08	−7.36	−8.33	−3.81	1
	ABUW_3830	ABUW_3830	UDP-glucose/GDP-mannose dehydrogenase	−6.71	−5.92	−4.92	−4.60	−1.72	1
	ABUW_1204	*trpG*	Para-aminobenzoate/anthranilate synthase glutamine amidotransferase component	−6.04	−4.45	−4.01	−3.72	−2.81	1
	ABUW_0332	ABUW_0332	FAD-linked oxidase domain protein	−6.02	−5.95	−5.30	−4.69	−3.23	1
	ABUW_3821	ABUW_3821	Spore coat polysaccharide biosynthesis protein spsC	−5.92	−7.59	−6.34	−5.32	−4.11	1
	ABUW_1110	*ddc*	Diaminobutyrate decarboxylase	−5.76	−4.56	−4.69	−6.22	−4.38	1
	ABUW_2357	ABUW_2357	Aspartate aminotransferase	−3.59	−2.80	−3.31	−3.10	−1.92	1
	ABUW_3448	ABUW_3448	Glycosyl transferase, group 1	−2.83	−5.76	−5.10	−3.83	−2.80	1
	ABUW_0106	*phoB*	Phosphate regulon transcriptional regulatory protein PhoB	−2.27	−1.53	−1.51	−2.03	−2.30	0
	ABUW_0643	*cysI*	Sulphite reductase	−2.18	−2.09	−1.84	−1.91	−1.47	0
	ABUW_2898	ABUW_2898	Hypothetical protein	−1.90	−1.71	−1.77	−1.43	−2.52	0
	ABUW_3054	*trxB1*	Thioredoxin-disulphide reductase	−1.88	−1.84	−1.59	−1.59	−1.42	0
	ABUW_3357	*ilvB*	Acetolactate synthase, large subunit	−1.79	−1.12	−1.08	−1.03	−2.90	0
	ABUW_0392	*recG*	ATP-dependent DNA helicase RecG	−1.67	−1.80	−1.68	−1.54	−1.32	0
	ABUW_3636	*hom*	Homoserine dehydrogenase	−1.39	−1.53	−2.18	−2.15	−5.01	1
	ABUW_0105	*phoR*	Phosphate regulon sensor kinase PhoR	−1.28	−1.18	−1.25	−1.15	−1.20	0
	ABUW_0250	*hisB*	Imidazoleglycerol-phosphate dehydratase	−1.11	−1.42	−1.20	−1.15	−3.77	0
Systemic infection only –blood, liver, spleen and kidneys (80 genes)	ABUW_3885	*purK*	Phosphoribosylaminoimidazole carboxylase, ATPase subunit	−9.65	−5.72	−4.58	−9.65	0.63	1
	ABUW_3824	ABUW_3824	Family 1 glycosyl transferase	−9.44	−9.44	−9.44	−9.44	−3.52	0
	ABUW_1537	*gidA*	Glucose inhibited division protein A	−8.74	−8.74	−6.64	−8.74	−1.94	0
	ABUW_1446	*purF*	Amidophosphoribosyltransferase	−8.48	−3.59	−3.71	−6.53	0.49	0
	ABUW_0059	*purC*	Phosphoribosylaminoimidazole-succinocarboxamide synthase	−8.32	−8.32	−8.32	−8.32	−3.23	0
	ABUW_1002	*purL*	Phosphoribosylformylglycinamidine synthase	−7.49	−7.83	−6.24	−3.88	0.37	0
	ABUW_3822	ABUW_3822	Bacterial transferase hexapeptide (three repeats) family protein	−6.62	−9.09	−9.09	−9.09	−4.16	0
	ABUW_2235	ABUW_2235	AFG1-family ATPase	−6.55	−3.58	−3.76	−3.64	−0.01	0
	ABUW_3829	ABUW_3829	Hypothetical protein	−6.54	−4.86	−3.81	−3.52	0.07	1
	ABUW_1228	*lipA1*	Lipoic acid synthetase	−6.53	−9.04	−6.14	−5.93	−2.78	1
	ABUW_2192	ABUW_2192	ABC-1 domain protein	−5.88	−4.18	−4.04	−3.63	−2.04	1
	ABUW_0980	*purN*	Phosphoribosylglycinamide formyltransferase	−5.85	−9.83	−9.83	−9.83	−0.42	1
	ABUW_0913	*pckG*	Phosphoenolpyruvate carboxykinase	−5.72	−5.67	−3.83	−3.76	−0.81	0
	ABUW_0262	ABUW_0262	ABC transporter ATP-binding protein	−5.37	−5.31	−5.13	−6.52	−0.77	1
	ABUW_3828	ABUW_3828	Hypothetical protein	−5.32	−5.24	−4.93	−4.98	−2.49	1
	ABUW_1012	ABUW_1012	ExsB protein	−5.26	−7.17	−4.62	−4.85	−0.26	1
	ABUW_1029	*lepA*	GTP-binding protein LepA	−4.97	−2.83	−3.55	−4.95	−0.94	1
	ABUW_1208	*ubiC*	Chorismate lyase	−4.96	−4.51	−3.77	−3.68	0.02	1
	ABUW_3833	*ptk*	Tyrosine-protein kinase ptk	−4.78	−4.08	−4.51	−5.53	−1.49	0
	ABUW_3260	ABUW_3260	Putative two-component response regulator	−4.72	−4.15	−3.41	−5.49	0.46	1
	ABUW_3651	ABUW_3651	Site-specific DNA-methyltransferase	−4.65	−4.24	−4.65	−4.29	−0.10	0
	ABUW_1221	ABUW_1221	Sensory box protein	−4.52	−4.10	−2.65	−3.04	−0.24	1
	ABUW_3689	*fadD*	Long-chain-fatty-acid--CoA ligase	−4.47	−3.12	−3.06	−3.17	0.21	0
	ABUW_3037	ABUW_3037	Hemerythrin/HHE cation-binding motif- containing protein	−4.41	−2.35	−1.87	−1.91	−0.40	0
	ABUW_2208	ABUW_2208	Adenylate/guanylate cyclase	−4.16	−6.00	−4.15	−4.19	−1.27	1
	ABUW_1022	*pabC*	Aminodeoxychorismate lyase	−3.94	−3.06	−3.39	−3.14	−0.29	0
	ABUW_1224	*ahcY*	Adenosylhomocysteinase	−3.90	−2.37	−3.03	−5.69	−0.47	1
	ABUW_2342	*ychF*	GTP-binding protein YchF	−3.65	−4.24	−3.30	−3.91	−1.30	1
	ABUW_1013	ABUW_1013	Radical SAM domain protein	−3.56	−5.16	−3.76	−5.30	0.04	1
	ABUW_0388	*corA*	Magnesium and cobalt transport protein	−3.51	−3.74	−3.02	−3.50	−0.74	1
	ABUW_1176	*bauB*	Ferric acinetobactin transport system periplasmic binding protein	−3.50	−3.84	−3.15	−2.37	−0.45	1
	ABUW_0719	*cafA*	Ribonuclease G	−3.46	−3.45	−3.06	−3.49	−0.41	1
	ABUW_0982	ABUW_0982	Permease	−3.23	−2.46	−2.85	−2.94	−0.47	0
	ABUW_3360	ABUW_3360	Hypothetical protein	−3.20	−3.44	−2.56	−2.49	−0.44	0
	ABUW_1177	*bauA*	Ferric acinetobactin receptor	−3.15	−3.27	−4.01	−4.74	−0.37	1
	ABUW_3642	ABUW_3642	Putative periplasmic carboxyl-terminal protease	−3.11	−4.73	−4.73	−2.81	0.94	1
	ABUW_1173	*bauD*	Ferric acinetobactin transport system permease	−3.09	−3.44	−3.26	−3.68	0.14	1
	ABUW_3246	*pabB*	P-aminobenzoate synthetase PabB	−3.08	−3.04	−2.82	−3.07	−0.42	1
	ABUW_0496	*ksgA*	Dimethyladenosine transferase	−3.08	−2.03	−2.10	−2.45	−1.12	0
	ABUW_0097	*serB*	Phosphoserine phosphatase	−3.05	−4.89	−5.19	−3.33	−2.75	1
	ABUW_3455	*truA*	tRNA pseudouridine synthase A	−3.05	−2.40	−2.27	−1.99	−0.61	0
	ABUW_3522	*recB*	Exonuclease V beta chain	−3.00	−3.04	−2.25	−2.84	−0.84	1
	ABUW_0533	*tgt*	Queuine tRNA-ribosyltransferase	−2.96	−1.71	−2.23	−2.34	0.39	0
	ABUW_3740	*znuA*	High affinity Zn transport protein	−2.94	−2.16	−2.28	−2.33	0.07	0
	ABUW_1000	*ruvA*	Holliday junction DNA helicase RuvA	−2.77	−3.33	−2.57	−2.95	−0.83	0
	ABUW_3743	*znuB*	High affinity Zn transport protein	−2.77	−2.19	−2.10	−2.53	−0.19	0
	ABUW_3447	*lpxL*	Lipid A biosynthesis acyltransferase	−2.65	−2.68	−2.76	−3.25	−0.02	0
	ABUW_1139	ABUW_1139	Hypothetical protein	−2.64	−2.01	−1.75	−1.33	0.05	0
	ABUW_3634	*xerD*	Tyrosine recombinase XerD	−2.59	−3.15	−2.30	−1.94	−0.05	0
	ABUW_3841	*rph*	Ribonuclease PH	−2.52	−2.21	−1.73	−2.05	−0.23	0
	ABUW_0549	ABUW_0549	Hypothetical protein	−2.41	−1.80	−1.77	−1.73	−0.64	0
	ABUW_3758	ABUW_3758	Protein DedA	−2.32	−2.08	−2.14	−2.14	−0.32	0
	ABUW_3369	ABUW_3369	Rhodanese domain protein	−2.27	−1.89	−1.75	−1.64	0.94	0
	ABUW_3741	*zur*	Transcriptional regulator, Fur family	−2.26	−1.72	−1.63	−1.50	0.48	0
	ABUW_0983	*hda*	DnaA family protein	−2.25	−1.69	−1.66	−1.46	−0.39	0
	ABUW_0898	*rrmJ*	Ribosomal RNA large subunit methyltransferase J	−2.21	−3.02	−2.31	−1.99	−0.95	0
	ABUW_0381	ABUW_0381	DEAD/DEAH box helicase	−2.19	−2.15	−1.41	−1.30	−0.18	1
	ABUW_3350	*prfC*	Peptide chain release factor 3	−2.13	−1.27	−1.31	−1.30	−0.48	0
	ABUW_0999	*ruvB*	Holliday junction DNA helicase RuvB	−2.11	−1.51	−1.91	−2.04	−0.83	0
	ABUW_0380	*suhB*	Inositol-1-monophosphate	−2.10	−2.14	−2.37	−2.89	−0.13	0
	ABUW_0724	ABUW_0724	Membrane protein involved in aromatic hydrocarbon degradation	−2.04	−2.23	−1.88	−1.53	−0.49	0
	ABUW_0281	*mraW*	S-Adenosyl-methyltransferase MraW	−2.02	−1.66	−1.61	−2.07	0.16	0
	ABUW_3401	*clpP*	ATP-dependent Clp protease, proteolytic subunit ClpP	−1.99	−2.91	−2.37	−2.87	−0.58	0
	ABUW_1072	*idh*	Isocitrate dehydrogenase, NADP-dependent	−1.96	−2.17	−1.79	−2.00	−0.38	1
	ABUW_0444	*rnr*	Ribonuclease R	−1.80	−1.58	−1.62	−1.74	0.06	0
	ABUW_3742	*znuC*	Zinc import ATP-binding protein ZnuC	−1.75	−1.33	−1.61	−1.85	0.51	0
	ABUW_0060	ABUW_0060	Hypothetical protein	−1.66	−1.89	−1.77	−2.12	−0.14	0
	ABUW_1220	ABUW_1220	Malate dehydrogenase	−1.66	−1.31	−1.15	−1.21	0.18	0
	ABUW_2971	*betB*	Betaine aldehyde dehydrogenase	−1.60	−1.16	−1.12	−1.13	−0.38	0
	ABUW_3513	*hslO*	Heat shock protein 33	−1.54	−1.54	−1.87	−1.46	0.10	0
	ABUW_3523	*recC*	Exonuclease V gamma chain	−1.53	−1.51	−1.78	−1.79	−0.58	1
	ABUW_3090	ABUW_3090	Hypothetical protein	−1.52	−1.79	−1.76	−2.00	0.27	0
	ABUW_0232	*trkD*	Potassium transport system low affinity (KUP family)	−1.49	−1.17	−1.21	−1.06	0.65	0
	ABUW_3521	*recD*	Exonuclease V alpha subunit	−1.39	−1.63	−1.84	−1.80	−0.65	0
	ABUW_1184	ABUW_1184	ABC transporter, ATP-binding protein	−1.35	−1.71	−1.70	−1.30	−0.68	0
	ABUW_1185	ABUW_1185	ABC transporter	−1.31	−1.46	−1.40	−1.50	−0.40	0
	ABUW_3529	*ubiB*	2-Octaprenylphenol hydroxylase of ubiquinone biosynthetic pathway	−1.23	−1.09	−1.01	−1.18	−0.26	0
	ABUW_1134	ABUW_1134	Major facilitator superfamily MFS_1	−1.21	−1.62	−1.58	−1.39	−0.10	0
	ABUW_3619	*phoU*	Phosphate transport system regulatory protein PhoU	−1.09	−1.45	−1.46	−1.26	0.20	0
	ABUW_0155	*aceE*	Pyruvate decarboxylase E1 component	−1.61	−1.61	−2.22	−1.88	1.72	0
Pneumonia infection – lungs only (67 genes)	ABUW_3247	*hisC*	Histidinol-phosphate aminotransferase	−0.71	−0.51	−0.62	−0.87	−5.43	1
	ABUW_1200	*trpC*	Indole-3-glycerol phosphate synthase	0.11	−0.11	0.00	−0.05	−5.03	1
	ABUW_3460	*leuB*	3-Isopropylmalate dehydrogenase	−0.30	−0.30	−0.23	−0.24	−3.88	0
	ABUW_3462	*leuD*	3-Isopropylmalate dehydratase, small subunit	−0.31	−0.37	−0.17	−0.07	−3.73	0
	ABUW_0617	*trpB1*	Tryptophan synthase, beta subunit	0.14	−0.13	−0.26	0.01	−3.63	0
	ABUW_2553	ABUW_2553	Hypothetical protein	−0.01	−0.11	0.06	0.54	−3.58	0
	ABUW_0249	*hisH*	Imidazole glycerol phosphate synthase, glutamine amidotransferase subunit	−0.26	−0.67	−0.51	−0.85	−3.57	0
	ABUW_2746	*hisZ*	ATP phosphoribosyltransferase regulatory subunit	−0.63	−0.59	−0.89	−1.14	−3.55	1
	ABUW_3356	*ilvN*	Acetolactate synthase, small subunit	−0.72	−0.84	−0.39	−0.27	−3.34	0
	ABUW_3249	*hisG*	ATP phosphoribosyltransferase	0.08	−0.55	−0.15	−0.62	−3.30	0
	ABUW_1201	*trpD*	Anthranilate phosphoribosyltransferase	0.71	0.14	0.42	0.34	−3.26	0
	ABUW_3406	*metX*	Homoserine O-acetyltransferase	0.01	0.02	−0.25	−0.06	−3.25	0
	ABUW_3531	*hisIE*	Phosphoribosyl (-ATP, -AMP) pyrophosphohydrolase/cyclohydrolase	−0.45	−0.88	−0.57	−0.62	−3.19	0
	ABUW_0246	*hisA*	Phosphoribosylformimino-5-aminoimidazole carboxamide ribotide isomerase	−0.05	−0.81	−0.25	−0.56	−3.10	0
	ABUW_3603	*trpE*	Anthranilate synthase component I	0.31	0.25	0.24	0.23	−3.10	0
	ABUW_1811	*argC*	*N*-Acetyl-gamma-glutamyl-phosphate reductase	−0.47	−0.13	−0.12	−0.25	−2.95	1
	ABUW_3463	*leuC*	3-Isopropylmalate dehydratase, large subunit	−0.26	−0.45	−0.37	−0.26	−2.81	0
	ABUW_0240	*hisF*	Imidazoleglycerol phosphate synthase, cyclase subunit	−0.09	−0.76	−0.90	−0.75	−2.77	0
	ABUW_0616	*trpF*	Phosphoribosylanthranilate isomerase	0.41	0.22	0.23	0.19	−2.75	0
	ABUW_0953	*lysA*	Diaminopimelate decarboxylase	0.32	0.19	−0.07	−0.04	−2.73	0
	ABUW_3302	*relA*	GTP pyrophosphokinase (ppGpp synthetase I)	0.74	0.34	0.37	0.72	−2.66	0
	ABUW_2440	ABUW_2440	Surface antigen	0.32	−0.23	−0.14	0.13	−2.48	0
	ABUW_0587	*ilvE*	Branched-chain amino acid aminotransferase	0.09	−0.16	0.15	0.12	−2.46	0
	ABUW_3355	*ilvC*	Ketol-acid reductoisomerase	−0.62	−0.43	−0.55	−0.22	−2.45	0
	ABUW_2150	*metZ*	O-Succinyl homoserine sulfhydrylase	0.24	−0.19	−0.05	−0.44	−2.33	0
	ABUW_0026	*ilvD*	Dihydroxy-acid dehydratase	−0.93	−0.67	−0.70	−0.55	−2.31	0
	ABUW_0622	*trpA*	Tryptophan synthase, alpha subunit	−0.76	−0.65	−1.04	−0.57	−2.28	0
	ABUW_1599	*metR*	Transcriptional regulator, LysR family	0.65	−0.08	0.12	0.27	−2.24	0
	ABUW_1274	ABUW_1274	Hypothetical protein	−1.28	−0.62	−1.19	−0.76	−2.12	0
	ABUW_3405	*leuA*	2-Isopropylmalate synthase	0.24	0.27	0.02	0.11	−1.99	0
	ABUW_1806	*ilvA1*	Threonine dehydratase	0.06	−0.16	−0.07	0.10	−1.99	0
	ABUW_2823	*argG*	Argininosuccinate synthase	−0.53	−0.57	−0.63	−0.76	−1.96	0
	ABUW_1732	*ntrC*	Nitrogen metabolism transcriptional regulator, NtrC, Fis Family	0.42	0.13	−0.12	−0.15	−1.93	0
	ABUW_3146	ABUW_3146	Putative ATP-binding protein	−0.98	−1.44	−0.95	−1.05	−1.92	0
	ABUW_0298	*gltB*	Glutamate synthase, large subunit	0.22	0.27	0.19	0.24	−1.86	0
	ABUW_2840	ABUW_2840	Lytic transglycosylase	−0.42	−0.64	−0.58	−0.42	−1.83	0
	ABUW_3272	*proC*	Pyrroline-5-carboxylate reductase	−0.87	−0.43	−0.48	−0.41	−1.82	0
	ABUW_3197	*metE*	5-Methyltetrahydropteroyltriglutamate- homocysteine methyltransferase	−0.80	−0.94	−1.16	−1.18	−1.79	0
	ABUW_3040	*argB*	Acetylglutamate kinase	−0.54	0.27	0.16	−0.05	−1.76	0
	ABUW_2250	*gspJ*	General secretion pathway protein J	−0.61	−0.12	0.28	−0.09	−1.61	0
	ABUW_0299	*gltD*	Glutamate synthase, small subunit	0.23	−0.10	−0.10	0.24	−1.57	0
	ABUW_3223	*argJ*	Glutamate *N*-acetyltransferase/amino-acid acetyltransferase	0.14	−0.08	−0.16	−0.34	−1.49	0
	ABUW_3407	*metW*	Methionine biosynthesis protein MetW	0.76	0.43	0.37	0.20	−1.48	0
	ABUW_3617	*argH*	Argininosuccinate lyase	−0.15	−0.65	−0.36	−0.48	−1.42	0
	ABUW_2063	ABUW_2063	Hypothetical protein	−0.32	−0.38	−0.49	−0.36	−1.42	0
	ABUW_1816	*aro1*	3-Deoxy-7-phosphoheptulonate synthase	−0.31	−0.36	−0.27	−0.31	−1.41	0
	ABUW_3198	ABUW_3198	Hypothetical protein	0.14	−0.36	−0.47	−0.58	−1.38	0
	ABUW_2658	ABUW_2658	Hypothetical protein	0.65	0.60	−0.05	0.25	−1.37	0
	ABUW_3408	ABUW_3408	Sel1 domain protein	−0.68	−0.78	−0.47	0.10	−1.36	0
	ABUW_2196	*alkR*	Transcriptional regulator, AraC family	0.06	−0.14	0.25	0.01	−1.33	0
	ABUW_1771	*pfkB*	1-Phosphofructokinase	−0.49	−0.47	−0.01	−0.45	−1.31	0
	ABUW_1118	*pstB*	Phosphate ABC transporter, ATP-binding protein	0.14	0.07	−0.02	0.08	−1.30	0
	ABUW_2094	ABUW_2094	Hypothetical protein	0.04	0.29	0.05	0.00	−1.27	0
	ABUW_1115	ABUW_1115	Phosphate ABC transporter, substrate-binding protein	0.04	−0.42	−0.17	−0.18	−1.22	0
	ABUW_1117	*pstA*	Phosphate ABC transporter, permease protein	−0.21	−0.25	−0.09	−0.27	−1.19	0
	ABUW_0690	ABUW_0690	ErfK/YbiS/YcfS/YnhG family protein	−0.73	−0.84	−0.59	0.79	−1.16	0
	ABUW_3389	*proA*	Glutamate-5-semialdehyde dehydrogenase	−0.59	−0.50	−0.46	−0.37	−1.16	0
	ABUW_3572	*fadB*	Fatty oxidation complex, alpha subunit FadB	0.38	−0.11	−0.03	0.20	−1.13	0
	ABUW_0031	*ppc*	Phosphoenolpyruvate carboxylase	0.06	0.09	0.14	0.22	−1.12	0
	ABUW_1715	ABUW_1715	Transcriptional regulator, GntR family	0.02	−0.14	0.05	−0.11	−1.12	0
	ABUW_1116	*pstC*	Phosphate ABC transporter, permease protein (EsvD)	−0.02	−0.04	−0.01	−0.10	−1.10	0
	ABUW_2234	*glcB*	Malate synthase G	−0.41	−0.39	−0.33	−0.32	−1.09	0
	ABUW_1733	*ntrB*	Signal transduction histidine kinase, nitrogen specific, NtrB	−0.17	−0.32	−0.02	−0.08	−1.09	0
	ABUW_1873	*pcaJ2*	3-Oxoadipate CoA-transferase subunit B	0.26	0.30	0.00	−0.08	−1.06	0
	ABUW_2146	*rnd*	Ribonuclease D	−0.16	−0.16	−0.14	−0.55	−1.04	0
	ABUW_1705	ABUW_1705	FAD-dependent pyridine nucleotide-disulphide oxidoreductase	0.10	−0.06	−0.05	−0.19	−1.01	0
	ABUW_0729	*uppP*	Undecaprenyl-diphosphatase UppP	0.02	−0.42	−0.17	−0.07	−1.01	0

*Genes previously identified as essential in at least one tissue (listed in Table 1), whereby a value of zero is non-essential and one is essential.

Of those genes identified as essential and/or required for fitness in all tissues, distinct biosynthetic and signalling pathways were identified. For example, *dat/ddc* are required for the synthesis of the polycationic polyamine, 1,3-diaminopropane (1,3-DAP) [43]. In contrast to other pathogens that synthesize and secrete larger polyamines, *Acinetobacter* only produces 1,3-DAP [44,46]. Given their role in diverse cellular functions, including mediating translation efficiency through RNA and ribosome binding, stabilizing dsRNA, promoting read through and initiation at non-optimal codons [47,49], modulating membrane permeability through OmpF/C binding [50], promoting phage resistance [51] and resistance to oxidative stress, it is unsurprising that their production and import are essential for virulence [52,55]. While the role of 1,3-DAP has yet to be fully elucidated in *A. baumannii*, it is known to modulate motility and biofilm formation [44,46] and is required for the biosynthesis of acinetoferrin, baumannoferrin and fimsbactin siderophores [56,58]. However, the functional significance of these siderophores in pathogenesis has yet to be confirmed, as to date only acinetobactin is required *in vivo* [58,59]. Similarly, to the best of our knowledge, the role of 1,3-DAP in the modulation of host immune responses has not been investigated. Given other polyamines have been shown to modulate pro-inflammatory cytokines, induce apoptosis and impair leucocyte chemotaxis [60,63], future research should explore this possibility.

The phosphate-sensing two-component regulatory system, PhoBR, was also required for fitness in all tissues. Given the role of these systems in cell envelope homeostasis, environmental protection and transcriptional regulation, it is unsurprising these are pivotal for fitness [8,64, 65]. While the mechanisms underlying this attenuation are unclear, the importance of this system is emphasized by the *pstABC* and *phoU*, encoding the phosphate uptake system and its regulator, respectively, also having significantly reduced fitness in the lungs and during septicaemia infection, respectively. Studies from other pathogens suggest that PhoBR may interact with other two-component systems, including BaeSR, which regulates maltose transport, chemotactic responses and flagellar biosynthesis in *E. coli*; outer membrane porin production in *Klebsiella pneumoniae*; and efflux pumps, Csu pili and phenylacetic acid catabolism in *A. baumannii* [66,68]. Furthermore, in *Serratia* spp. PhoB regulates quorum sensing and the transcriptional regulator, Rap, homologous to that of *Yersinia* spp. RovA, which regulates genes associated with host colonization [69]. While no fitness defects were observed for the *baeSR* mutants in this study, these data highlight the importance of delineating the PhoBR regulon in *A. baumannii*, given the potential of its role in virulence regulation over and above its function in cell homeostasis.

### Genes specific to pneumonia or septicaemia infection

**Fig. 2.**
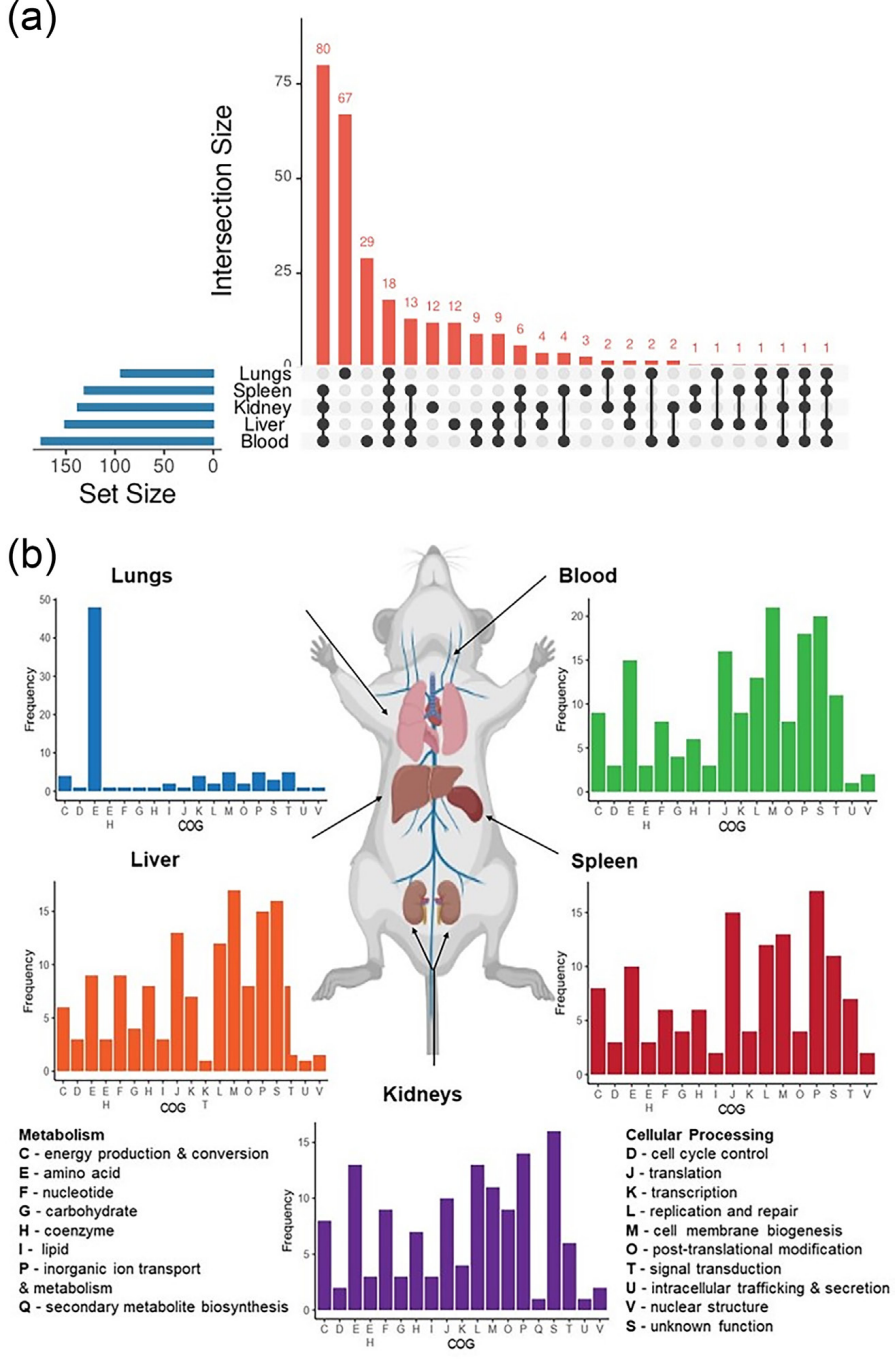
Genes required for *in vivo* fitness. (**a**) The distribution of the 281 genes for which transposon insertions significantly impact bacterial fitness (log_2_ fold change < −1 and FDR<0.01) during *in vivo* colonization of various tissues. UpSet plot (generated using https://asntech.shinyapps.io/intervene/) highlights the overlapping subset of genes associated with fitness in multiple tissues. Individual gene locus tags represented in the UpSet plot are provided in Table S7. A total of 18 genes were identified as important for fitness in all tissues, irrespective of infection route, and 80 and 67 genes were important for fitness specifically in septicaemia and pneumonia infections, respectively. (**b**) Tissue specificity and COG categories required for *in vivo* fitness in the blood, liver, kidneys, spleen and lungs. The COG category for the genes in which transposon insertions significantly impact fitness was determined by EggNOG Mapper. Categories represented within the dataset include those associated with energy production and conversion (**C**), cell cycle control and mitosis (**D**), amino acid metabolism and transport (**E**), nucleotide metabolism and transport (**F**), carbohydrate metabolism and transport (**G**), coenzyme metabolism (**H**), lipid metabolism (**I**), translation (**J**), transcription (**K**), replication and repair (**L**), cell wall/membrane/envelop biogenesis (**M**), post-translational modification, protein turnover and chaperone functions (**O**), inorganic ion transport and metabolism (**P**), secondary structure (**Q**), function unknown (**S**), signal transduction (**T**), intracellular trafficking and secretion (**U**) and nuclear structure (**V**). The tissues examined during septicaemia infection consistently showed increased representation of genes associated with transcription, post-transcriptional modification, signal transduction and intracellular trafficking, while genes associated with amino acid metabolism and transport were important for bacterial fitness in the lungs.

A total of 67 and 80 genes were specifically required for fitness during pneumonia and septicaemia infections, respectively (including 28 and 4 previously identified as essential, Table 2, Fig. 2a and Table S7). To identify functional groups linked to specific disease states, mutants with reduced *in vivo* fitness were analysed based on clusters of orthologous gene (COG) categories (Fig. 2b and Table S6).

The most pronounced functional group associated with pneumonia was amino acid metabolism and transport, with 39 of the 67 genes identified associated with these pathways. Similar to most pathogens, *A. baumannii* encodes multiple systems for amino acid biosynthesis and catabolism and can utilize these as carbon or nitrogen sources [70,72]. While the lungs contain an array of amino acids in micromolar concentrations, biosynthetic pathways, particularly those associated with branched and aromatic amino acids, are known to be essential for the survival of *A. baumannii*, *K. pneumoniae* and to a lesser extent *S. aureus* [20,70, 73,75]. It has previously been postulated that these available amino acids serve as a nutrient source during infection [70]. Apart from nutrient sources, amino acid metabolism may impact virulence in other ways. For example, the catabolism of histidine is important for the release of zinc during starvation [70,76]. Histidine can also be degraded to histamine, which cannot be utilized by *Acinetobacter*, however, is required for acinetobactin synthesis and can modulate interactions with neutrophils by reducing phagocytosis [58,72, 77]. Therefore, while the mechanistic details remain to be elucidated, these data do highlight the unique requirements of *A. baumannii* during pneumonia infection.

Interestingly, in contrast to the findings described above, the purine metabolism genes (*purCDEHU*) were not required for fitness during pneumonia infection. While this observation is in conflict with that reported in the literature [20], closer inspection of our dataset revealed that the read counts (c.p.m.) associated with these genes were lower in the pneumonia doses compared to the septicaemia doses. This likely compromised the statistical power and hindered our ability to accurately define fitness defects within this gene cluster. Highlighting that our dataset is not exhaustive, and given the specific experimental conditions, it is plausible that additional pathways not identified here may also contribute.

In terms of septicaemia and disseminated infection, the cell membrane and biogenesis functional group had the highest representation, with the exception of the kidneys, where genes of unknown function were most prevalent. Interestingly, translation, replication and repair, inorganic ion transport and metabolism were all highly represented in the tissues involved in septicaemia and disseminated infection (>10 genes per category), with signal transduction also overrepresented in the blood. Notably, the disruption of metal uptake genes was only impaired for fitness during septicaemia. During infection, the host restricts nutrients and metals through a process termed ‘nutritional immunity’ [78]. Iron, zinc and manganese are biologically important and required for numerous cellular processes, including transcription, amino acid synthesis, metabolism, pH balance, DNA replication and repair [59,78,82], and the ability of *A. baumannii* to acquire these is a critical mediator of pathogenesis [72]. As expected, genes associated with acinetobactin uptake (*bauABCDE*) were either essential for survival in septicaemia (*bauABCD*) or had significantly reduced fitness (*bauE,* blood and spleen) [58].

The zinc regulator and uptake genes (*zur* and *znuABC*) were also identified during septicaemia infection. While *znuAB* are known to be transcriptionally upregulated in the lungs [78], our data show that their disruption only impacts fitness during systemic infection [79,83]. Consistent with this, others have shown that the inhibition of the haem uptake system via Gallium protoporphyrin IX (in LAC-4) and the disruption of the zinc-regulated lipoprotein, ZrlA (in ATCC17978), induced fitness defects during systemic dissemination from a lung infection [84,85]. Taken together, these data show that *A. baumannii* utilizes specific genes and pathways during *in vivo* infection that are infection syndrome specific. Such insights may provide the foundation for future therapeutics that are targeted for a given bacteria (in this case *A. baumannii*) and infection syndrome.

### Using Tn-Seq to examine organ specificity

**Fig. 3.**
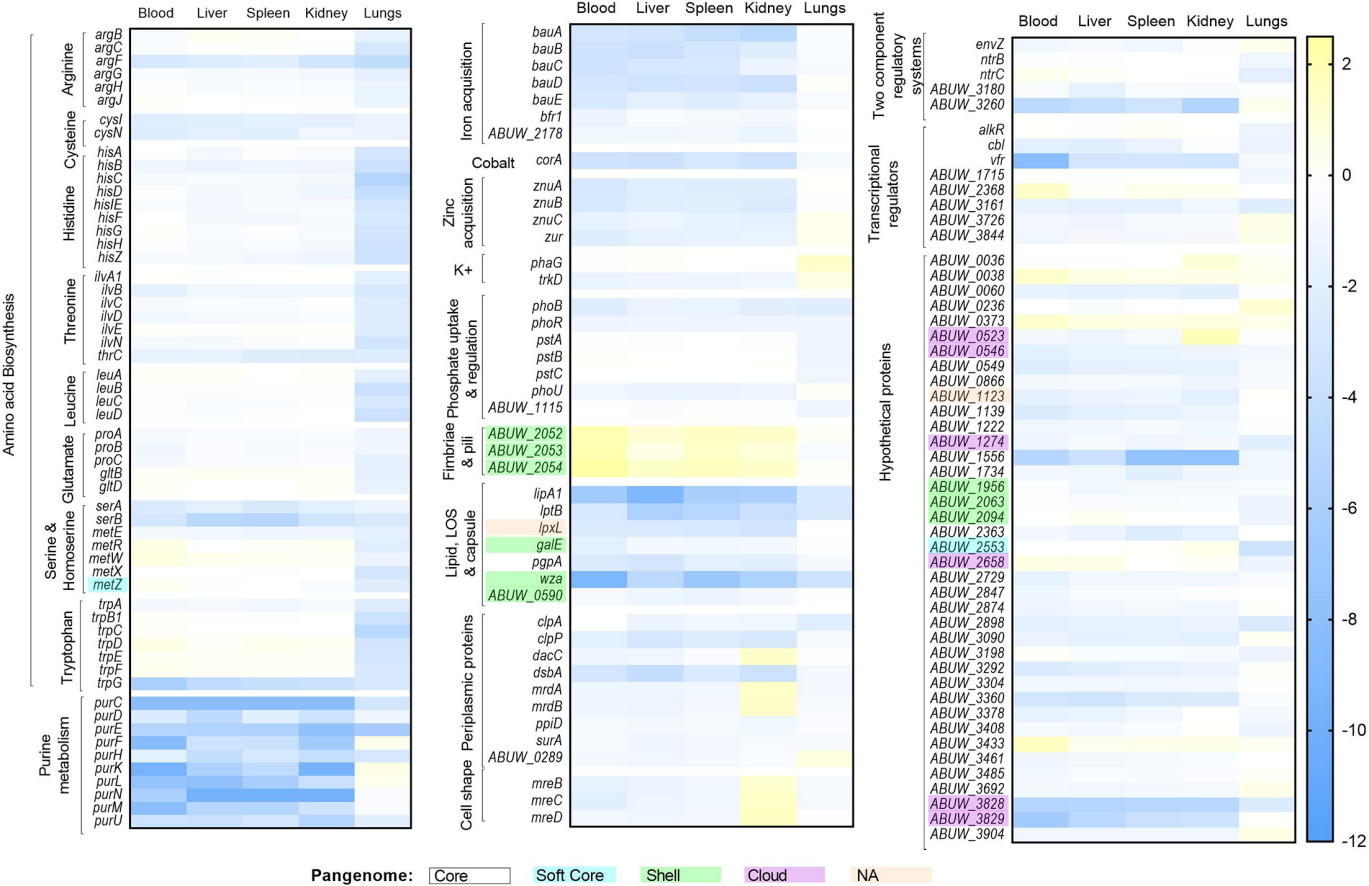
Tissue specificity and functional groupings of selected genes shown to have aberrant fitness *in vivo*. Heatmap representation of the log_2_ fold change in reads obtained for respective tissues relative to the input pool [intraperitoneal (IP) input for blood, liver, spleen and kidneys and intranasal (IN) input for lungs]. Where individual genes are denoted by numbers, these refer to their corresponding ABUW_locus tag. Genes with reduced transposon insertions (important for maximal fitness) are shown in shades of blue, while genes with increased numbers of insertions (increased fitness) are shown in yellow. Genes have been grouped based on functional category and cellular pathway. With gene names and locus tags highlighted in accordance with their pangenome designation: core (white), soft core (turquoise), shell (green), cloud (purple) and unassigned denoted as NA (orange). Genes associated with lipid, lipooligosaccharide (LOS) and capsule biosynthesis (*lipA*, *lptB* and *wza*) show a largely consistent reduction in reads across all tissues, while transposon insertions in amino acid biosynthesis genes have significantly reduced reads in the lungs during pneumonia infections but mostly show little impact on fitness in other tissues. Fimbriae and pili-associated genes have increased numbers of reads during septicaemia infection, with largely nonsignificant differences observed during pneumonia infection. While differences in infection route showed the most significant separation of the data, tissue specificity was also observed for genes with both increased and decreased fitness, as shown by the *mreBCD* gene cluster in the kidneys and *galE* in the blood, respectively.

Given the diversity of environments encountered during infection, we evaluated the contribution of individual genes to tissue specificity (Fig. 3). Examination of genes uniquely impacting fitness in the blood identified 19 genes with impaired fitness that encoded for 4 hypothetical proteins, 2 GGDEF family proteins, 6 transcriptional regulators and RNA-associated genes, 2 UDP-glucose 4-epimerases, the exopolyphosphatase PPX*,* bacterioferritin, Bfr1 and heat shock protein, HtpX. Bloodstream infection remains one of the most life-threatening forms of *A. baumannii* infection, and the role of these proteins in this infection syndrome needs further investigation. While bacterioferritin may denote a niche specific iron utilization pathway within the blood compartment [86], the presence of *ppx* in this group is surprising given its role in maintaining intracellular polyphosphate and contribution to virulence [87,88]. Of the six genes uniquely associated with fitness in the liver, two were hypothetical proteins, two periplasmic proteins (SurA and penicillin binding protein 1A), one transcriptional regulator and the sensor kinase, BfmS. While several studies have investigated the regulon of BfmRS highlighting its role in cell homeostasis, its contribution to virulence remains inconclusive [20,89, 90]. Five genes were uniquely associated with aberrant fitness in the kidneys, including two genes that were encoded for hypothetical proteins, one a YCII family containing protein, one tRNA hydrolase and the periplasmic chaperone, *ppiD*. While the overexpression of PpiD is known to compensate for the loss of the periplasmic chaperones *surA/skp* in *E. coli*, its protein specificity has yet to be confirmed in *A. baumannii* [91,92]. Although it is tempting to speculate that this may indicate a kidney-specific PpiD substrate, this requires further investigation. Finally, in the spleen, only three genes were identified with impaired fitness, but all of them had reduced fitness in other tissues that were not accompanied by significant FDR values, suggesting that despite the differences in tissue microenvironments, clear overlap exists.

### Gene disruptions with enhanced *in vivo* fitness

To determine whether there were any gene disruptions that led to enhanced fitness *in vivo*, and the tissue tropism of that effect, we applied a similar analysis that focused on genes with a log_2_ fold change >1 (FDR<0.01). A total of 28 mutants with enhanced fitness were identified (Table 3). Four mutants showed enhanced fitness during a disseminated septicaemia infection, including three that were fimbrial genes, encoding a fimbrial usher and subunit and a pili assembly chaperone. These findings are supported by a previous report*,* whereby loss of the type IVa pili and flagellin components was positively selected for during *P. aeruginosa* infection, a finding attributed to their role in innate immune activation [93]. Twenty-four genes were tissue specific, including eight, nine and seven genes that had increased fitness in the blood, kidneys and lungs, respectively. For the blood, the eight genes encoded three hypothetical proteins, two GGDEF family proteins, two transcriptional regulators and the penicillin-binding protein carboxy-terminal protease, *prc*. Six of the nine genes specifically associated with increased fitness in the kidneys (*dacC*, *mrdAB* and *mreBCD*) encoded penicillin-binding proteins and cell shape regulators [94,97]. Previous studies have proposed that bacterial cell shape and size are related to fitness and have an inverse relationship with phagocytosis [98,100]. Of the seven genes with elevated fitness in the lungs, four were linked to energy production and catabolism (*gabT*, *aceE*, *dadA2* and *hutU*). While this may reflect the altered nutrient availability in the lung environment, it is tempting to speculate that these observations may be linked to the modulation of the host immune response. For example, GabT is responsible for the metabolism of gamma-aminobutyric acid (GABA) [101]. GABA is also a potent signalling molecule, capable of modulating cytokine secretion, cell proliferation, phagocytic activity and chemotaxis [102,103]. These data provide important tissue-specific findings of enhanced bacterial fitness during *in vivo* infection and provide insights into potential new therapeutic targets and host-pathogen mechanisms, warranting further investigation.

**Table 3.** Gene disruptions resulting in elevated bacterial fitness in at least one tissue

Tissue specificity	Locus tag	Gene name	Known or predicted function	Transposon insertion rate fold change (log_2_)				
				Blood	Liver	Spleen	Kidney	Lung
All tissues	ABUW_0188	ABUW_0188	GGDEF family protein	2.26	1.08	1.34	1.22	1.24
Blood, liver, spleen and kidneys	ABUW_2054	ABUW_2054	Outer membrane fimbrial usher protein	2.38	1.39	1.67	1.37	0.12
Blood, spleen and kidneys	ABUW_2052	ABUW_2052	Fimbrial subunit	2.02	1.15	1.57	1.36	0.16
Blood and spleen	ABUW_2053	ABUW_2053	Pili assembly chaperone	2.07	0.70	1.41	1.02	−0.44
Blood only	ABUW_3433	ABUW_3433	Hypothetical protein	1.62	0.36	0.50	0.66	0.27
	ABUW_2368	ABUW_2368	Transcriptional regulator, LysR family	1.33	0.13	0.44	0.39	−0.12
	ABUW_0038	ABUW_0038	Hypothetical protein	1.30	0.69	0.48	0.59	0.47
	ABUW_3471	ABUW_3471	Hca operon transcriptional activator	1.28	0.83	0.79	0.81	0.75
	ABUW_0373	ABUW_0373	Hypothetical protein	1.26	0.75	0.66	0.82	0.54
	ABUW_3385	*prc*	Carboxy- protease	1.24	−0.12	−0.17	0.13	0.30
	ABUW_1045	ABUW_1045	GGDEF family protein	1.23	0.87	0.95	0.83	−0.13
	ABUW_0506	ABUW_0506	GGDEF domain protein	1.16	0.81	0.81	0.83	−0.03
Kidneys only	ABUW_0523	ABUW_0523	Hypothetical protein	−0.81	−1.30	−0.73	1.76	−0.25
	ABUW_0717	*mreD*	Rod shape-determining protein MreD	−0.95	−1.03	−0.89	1.53	−0.35
	ABUW_0716	*mreC*	Rod shape-determining protein MreC	−2.09	−1.31	−1.00	1.44	−0.68
	ABUW_2876	*mrdA*	Penicillin-binding protein 2	−1.23	−1.21	−0.98	1.42	−0.77
	ABUW_0524	ABUW_0524	YCII-related protein	−0.18	−0.17	−0.10	1.38	0.30
	ABUW_1244	*mrdB*	Rod shape-determining protein RodA (EsvE3)	−1.19	−1.33	−0.82	1.35	−0.59
	ABUW_1127	*dacC*	Penicillin-binding protein 6 (d-alanyl-d-alanine carboxypeptidase)	−1.16	−1.02	−0.39	1.34	−0.21
	ABUW_0715	*mreB*	Rod shape-determining protein MreB	−1.67	−1.52	−1.04	1.25	−0.66
	ABUW_0036	ABUW_0036	Hypothetical protein	−0.22	−0.16	−0.01	1.10	0.36
Lungs only	ABUW_0155	*aceE*	Pyruvate decarboxylase E1 component	−1.61	−1.61	−2.22	−1.88	1.72
	ABUW_0203	*gabT*	4-Aminobutyrate transaminase	−0.01	0.06	−0.01	−0.02	1.31
	ABUW_3539	*phaG*	pH adaptation potassium efflux system protein G	−0.29	0.14	0.02	−0.05	1.31
	ABUW_0667	ABUW_0667	Activator of HSP90 ATPase	0.63	−0.08	−0.01	0.00	1.14
	ABUW_0236	ABUW_0236	Hypothetical protein	−0.10	−0.44	−0.09	−0.11	1.14
	ABUW_3789	*dadA2*	d-Amino acid dehydrogenase small subunit	0.15	0.07	0.03	0.24	1.13
	ABUW_0077	*hutU*	Urocanate hydratase	0.02	0.11	0.15	0.21	1.09

### Conservation of genes required for *in vivo* fitness and correlation with other strains and infection models

Given the genetic diversity of *A. baumannii*, we assessed the conservation of the identified infection-related genes across different genetic lineages, using a limited pangenome to confirm these were not restricted to particular clonal types. Due to the potential for erroneous gene absences in genome assemblies containing contig breaks, only the 172 closed *A. baumannii* genomes on the NCBI (as of March 2020) were used to construct the pangenome and identify species-specific core or accessory genes. The pangenome contained 14,333 genes, which were designated core (present in >98% of genomes, *n*=2,577), soft-core (95–98%; *n*=169), shell (15–95%; *n*=1589) or cloud genes (<15%; *n*=9998). Previous *A. baumannii* pangenome studies have calculated a core genome of ~2,200–2,500 genes and a pangenome of ~19–20,000 genes total [104,105]. Comparison of genes with aberrant fitness *in vivo* to the *A. baumannii* pangenome identified that 89% (270/302) were part of the core genome, while 7, 13 and 9 genes were assigned to the soft-core, shell and cloud, respectively (Fig. S4). While the majority were present in International clonal (IC) type I, II and non-clonal type strains, four capsule locus genes were highly restricted, including ABUW_3828 and ABUW_3829 that were present in only five strains. This was in addition to the glycosyltransferase and hexapeptide transferase (ABUW_3824 and ABUW_3822) present in three and nine strains, respectively. As expected, none of the fitness genes were restricted to IC type I or II (Table S8).

Given the heterogeneity of disease observed with different animal models and *A. baumannii* strains, we sought to compare our fitness data with that previously published. Of the 302 fitness-associated genes identified in our dataset, 22 and 88 were previously identified as essential for survival in *Galleria* or during pneumonia infection with AB5075 and ATCC17978, respectively [17,20]. More than half (50/88) of the genes previously found to be required for survival in the lungs were consistent with our dataset. However, a comparison across all of the three studies revealed that only eight genes were shared, emphasizing the impact of different bacterial strains and infection models.

### Experimental validation using single-gene disruption mutants

**Fig. 4.**
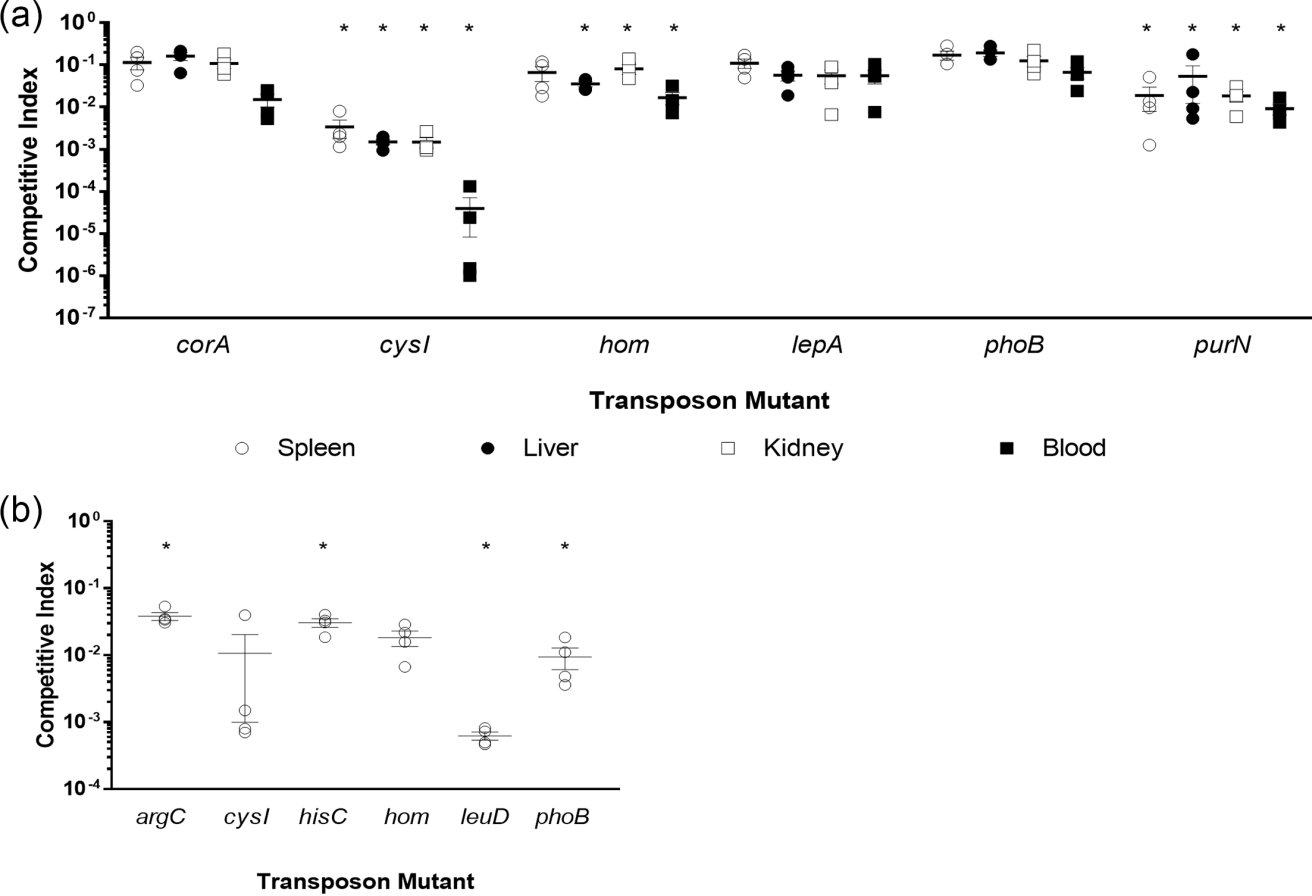
Validation of *in vivo* phenotypes. *In vivo* validation of the mutants with attenuated fitness during (**a**) septicaemia or (**b**) pneumonia infections. Mice were infected with an equal number of wild-type and mutant bacteria with a total inoculation dose of 5×10^4^ c.f.u. (intraperitoneal) or 1×10^8^ c.f.u. (intranasal). Tissue samples were recovered at 8 h post-infection and competitive indices determined; a competitive index <1 indicated a fitness defect. Each data point represents an individual animal, with four mice per group and experiments conducted on two separate days for each infection route. Mean and standard error are represented by the horizontal and vertical lines. Statistically significant differences in bacterial colonization were determined using Mann–Whitney, *P=<0.05 (recovered c.f.u. values available in Fig. S6).

To validate these data, we selected genes that were either essential for survival or had significantly reduced bacterial fitness. Our validation set included genes required for infection irrespective of the route (*cysI*, *hom* and *phoB*) and genes specifically required for either pneumonia (*argC*, *hisC* and *leuD*) or septicaemia (*corA*, *lepA* and *purN*). The respective mutants were obtained from the ordered AB5075-UW mutant library (Table S1) and were confirmed by PCR and southern hybridization (Fig. S5). The *in vitro* growth rates and levels of capsule and poly-*β*-(1–6)-*N*-acetylglucosamine production were comparable between all the mutants and the WT strain, while *in vitro* competitive fitness assays verified there were no significant growth defects (Fig. S6A–C). Using a competitive infection model (consistent with our Tn-Seq experiments), all mutants displayed a fitness decrease relative to the WT in all tissues tested (Fig. 4). While a statistically significant reduction in fitness was only observed in two (*cysI* and *purN*) and four (*argC*, *hisC*, *leuD* and *phoB*) of the mutants tested in our septicaemia and pneumonia models, respectively (*P* = < 0.05, c.f.u. values reported in Fig. S7), this was likely due to variability in the levels of colonization between different mice. However, the trend of reduced fitness was consistent with our initial observations, highlighting the enhanced sensitivity that Tn-Seq can offer.

### The limitations of this approach

While our dataset provides an extensive global overview of the genes and cellular components required for *in vivo* fitness, it is not without limitations. Firstly, our study does not account for the impact of host-derived metabolites. For example, extracellular metabolites including IMP, AMP and GMP (components of purine biosynthesis/salvage pathway) change dynamically in BAL fluid during the course of infection in response to lung injury and inflammation [106], and while their accumulation may occur at different rates, accounting for differences between datasets, this highlights the dynamic host-pathogen interplay. Secondly, the nature of TraDIS and population-based Tn-Seq experiments fails to account for potential transposon-mediated polar effects, the impact of contamination and/or population ‘cheats’, whereby specific mutants, otherwise nonviable in monoculture, persist by exploiting resources secreted by neighbouring cells [107,108]. The potential for these occurrences underscores the critical need for rigorous validation and comprehensive characterization studies, incorporating complemented mutants. Furthermore, although the methods employed (i.e., PCR-based library enrichment) were designed to selectively enrich for *A. baumannii*, the potential influence of contamination and co-culture dynamics on mutant survival cannot be excluded in the absence of monoculture validation experiments, which were beyond the scope of this study.

## Conclusions

The rapid spread of antibiotic-resistant Gram-negative bacteria is considered one of the greatest threats to modern medicine. Accordingly, the WHO has classified carbapenem-resistant *A. baumannii* as the top priority pathogen, urgently requiring novel therapeutics [7,109]. Compared with other Gram-negative pathogens, *A. baumannii* research has lagged with vast portions of the genome still uncharacterized and the mechanisms responsible for pathogenesis still to be confirmed. To address the current void, a more complete understanding of the genes required for *A. baumannii* infection is required. This study provides a comprehensive unbiased *in vivo* assessment of the genes required for *A. baumannii* survival and fitness during pneumonia and septicaemia infection. Our results provide the first indication of genes and pathways specifically required for infection in different tissues and provide early insights into the design of potential therapeutics to combat this dangerous pathogen.

This study has identified the important contribution of 38 hypothetical proteins, highlighting the urgent need for further research to characterize their function. Our dataset highlights the primary importance of the *A. baumannii* core genome in the context of infection, whereby 89% of the 302 genes with altered fitness were part of the core genome and present in >98% of strains, alluding to conserved mechanisms of bacterial fitness during infection. Furthermore, this work has provided the first unique opportunity to explore the requirements of bacterial genes across individual host tissues, highlighting the interdependence of many pathways, despite the diverse environments. Importantly, we were also able to validate the *in vivo* findings for representative infection-related genes, providing potential targets and pathogenic mechanisms of *A. baumannii* disease that are ripe for further characterization.

## Supplementary material

10.1099/mgen.0.001556 Uncited Supplementary Material 1.

10.1099/mgen.0.001556 Uncited Supplementary Material 2.

## Abbreviations

COG: clusters of orthologous gene
1,3-DAP: 1,3-diaminopropane
FDR: false discovery rate
LB: Luria-Bertani
