# Supplementary material for: Gene dependence during mammalian Acinetobacter baumannii pneumonia and septicaemia infections
Source: Microb Genom. 2025 Nov 11;11(11):001556. doi: 10.1099/mgen.0.001556 (PMC12604733; doi:10.1099/mgen.0.001556)
Supplement: Uncited Supplementary Material 1. [file mgen-11-01556-s001.pdf]

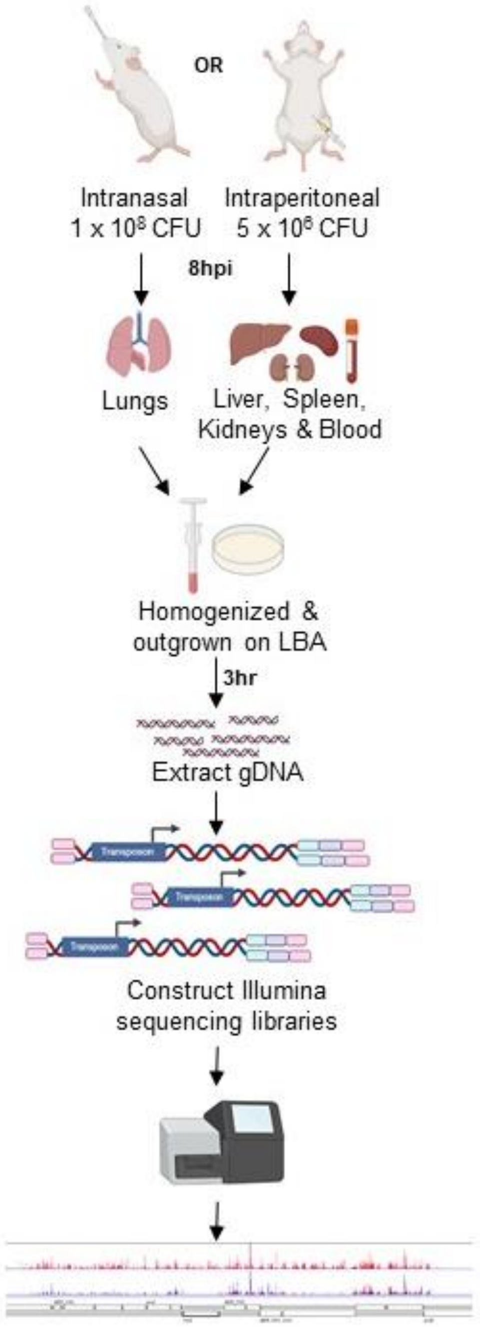

Supplementary Figure 1. Experimental design to assess the genes required for *in vivo* fitness. Immunocompetent, 6–8-week-old female BALB/c mice were infected with the *A. baumannii* strain AB5075-UW transposon mutant pool via the intranasal (1×10^8 CFU) or intraperitoneal (5×10^6 CFU) route. Mice were euthanized eight hours post infection and either the lungs (intranasal only) or liver, spleen, kidneys and blood (intraperitoneal) collected. Tissues were homogenized in phosphate buffered saline (PBS) and blood was mixed with an equal volume

of PBS, prior to plating across 10 lysogeny broth (LB) agar plates (without selection) and outgrown for three hours at 37°C. Bacteria were recovered from each plate, washed twice with PBS and DNA extracted using QIAGEN DNeasy blood and tissue kit. Illumina-compatible sequencing libraries were generated for each DNA sample (from every tissue and every mouse individually) using the terminal deoxynucleotidyl transferase (TdT) method, incorporating index sequences and Illumina-specific adaptors. Prior to sequencing, on an Illumina MiSeq, equal molar concentrations of each individual library were pooled to generate a single output for each tissue per biological repeat. Sequence reads were trimmed and aligned to the genome of *A. baumannii* strain AB5075-UW for comparison between different tissues and infection routes.

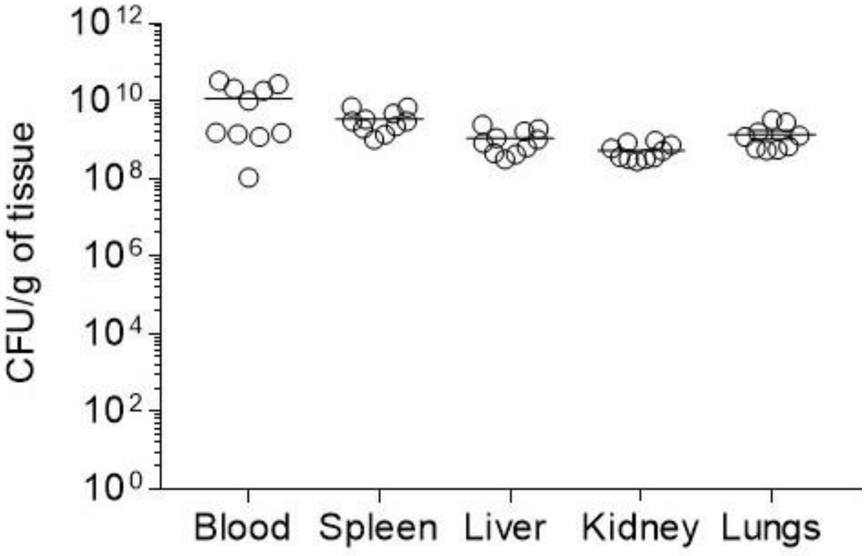

Supplementary Figure 2. Bacterial colonization eight hours post infection with the AB5075-UW transposon library via intraperitoneal (blood, spleen, liver, and kidneys) or intranasal (lungs only) routes with 5×10^6 CFU or 1×10^8 CFU per dose, respectively. Each data point represents an individual animal, with experiments conducted on two separate days for each infection route, n = 9 (lungs only) or n = 10 (all remaining tissues). Data represents CFU/gram of tissue or mL of blood as determined prior to outgrowth.

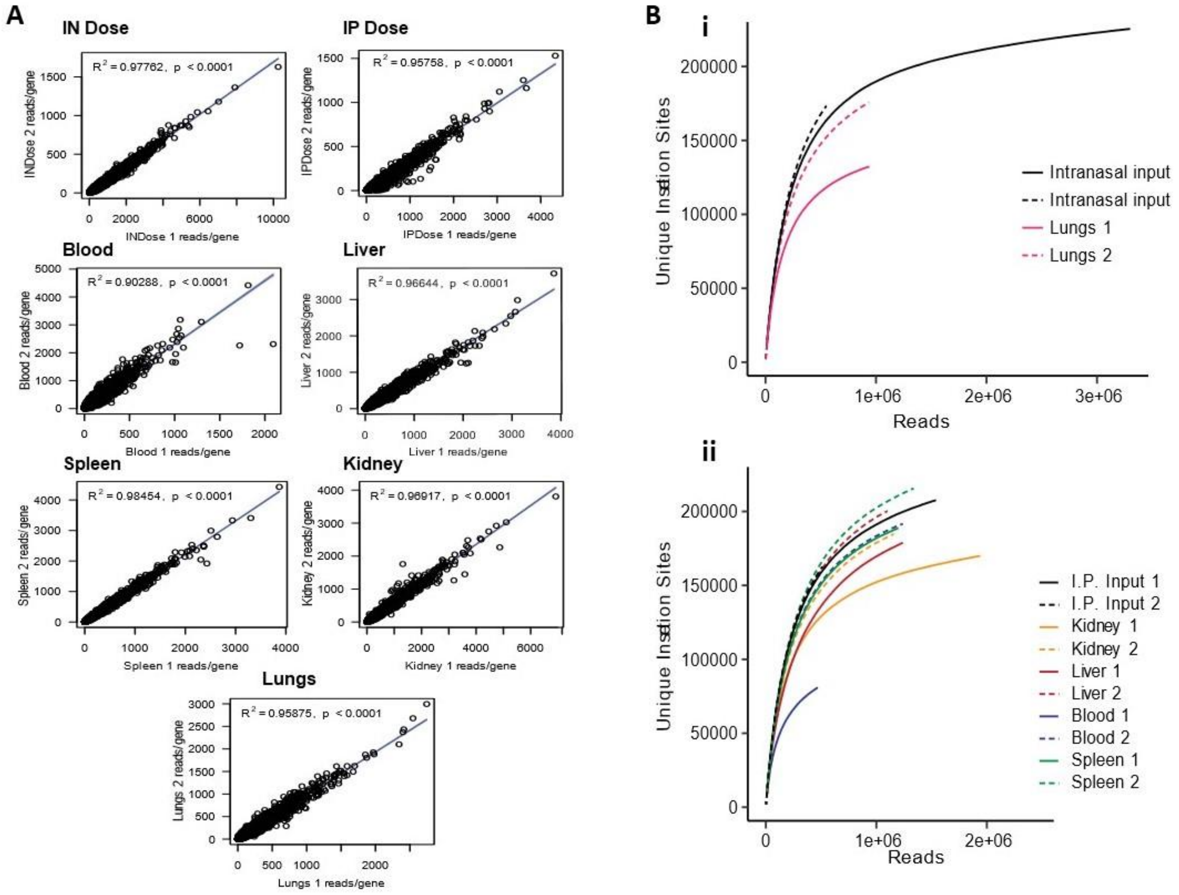

Supplementary Figure 3. Correlation of sequencing data between biological replicates. (A) Correlation of transposon insertion site reads per gene for samples from separate biological replicates performed on different days. IN dose refers to intranasal infection dose, IP dose refers to intraperitoneal dose, 1 and 2 refer to the separate biological replicates performed on different days. (B) Sequencing saturation for each sample, where 1 and 2 denote separate biological replicates. Panel i, intranasal infections; panel ii, intraperitoneal infections.

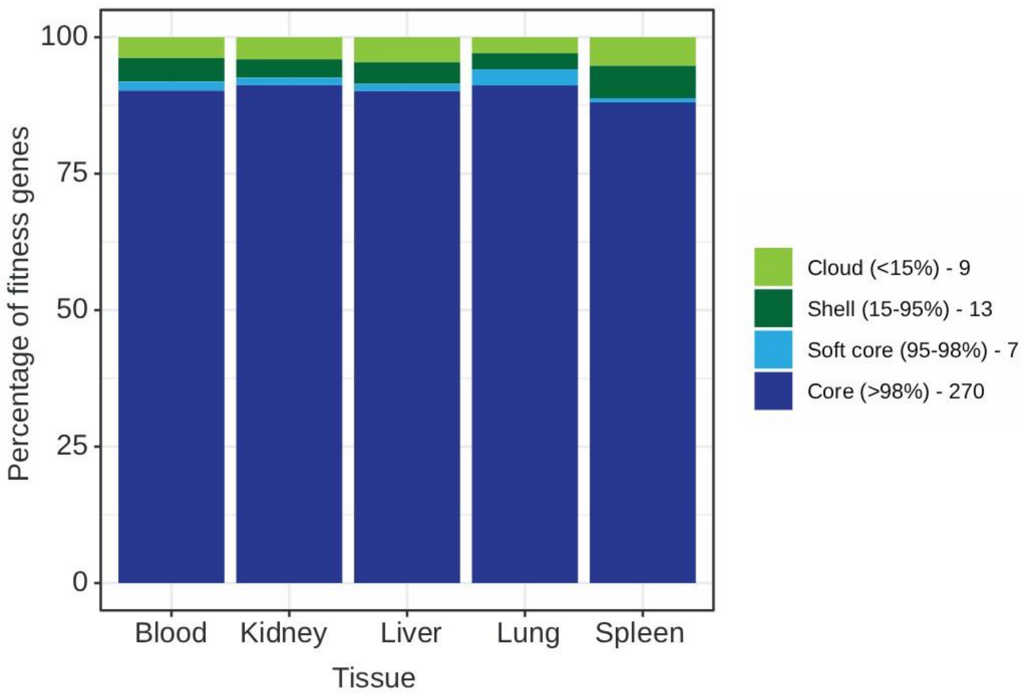

Supplementary Figure 4 - Conservation of genes associated with bacterial fitness. Pangenome analysis of genes required for *in vivo* fitness, when comparing these to 172 closed *Acinetobacter baumannii* genomes. The percentage used to define each category is provided in brackets and the exact number of genes assigned to each category is denoted after the hyphen. As expected the majority (270 of 302) of genes belong to the core genome, while a small percentage of genes required for bacterial fitness in each tissue are associated with the cloud (9 of 302) and shell (13 of 302), indicating potential genes which are strain specific. Of note, three genes (ABUW_1045, ABUW_1123 and ABUW_3347) were not assigned to any category.

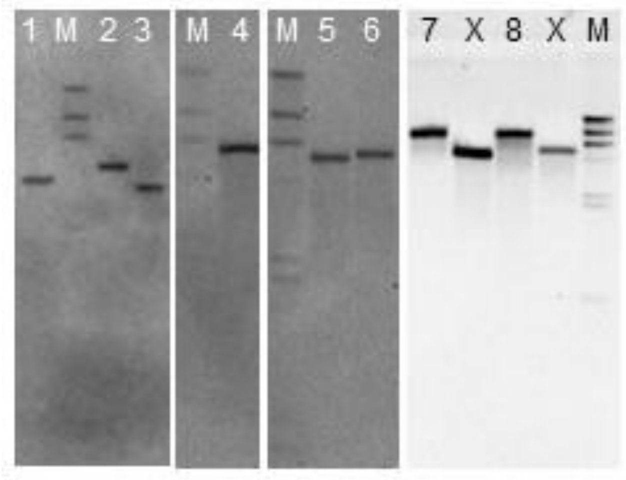

Supplementary Figure 5. Genetic validation of single *A. baumannii* AB5075-UW transposon mutants by Southern blot hybridization using a DIG-labelled tetracycline-specific probe, detected using Anti-DIG Fab fragments (Sigma) and CDP star (Roche) detection solution, to confirm the absence of secondary transposon insertions. Lanes contain equal concentrations of genomic DNA digested with a combination of either *Bgl*II, *Mfe*I, *Pvu*I, *Pvu*II, *PflM*I, *Sca*I, and/or *Spe*I, separated by gel electrophoresis through 1.2% (w/v) agarose in Tris-Acetate EDTA (TAE); AB5075-UW transposon mutants 1. *phoB* (6695 bp), 2. c*orA* (4365 bp), 3. *purN* (3458 bp), 4. *lepA* (5889 bp), 5. *argC* (5376 bp), 6. *hisC* (5601 bp), 7. *hom* (8197 bp), 8. *cysI* (8428 bp), M. refers to Roche DNA molecular weight marker II. X denotes other mutants not described in this study. Only single insertions were detected in the mutants and no fragments were detected in wild-type AB5075-UW genomic DNA digested with the same combination of enzymes and tested in parallel (data not shown), consistent with the absence of a transposon insertion and the tetracycline sensitive nature of this strain.

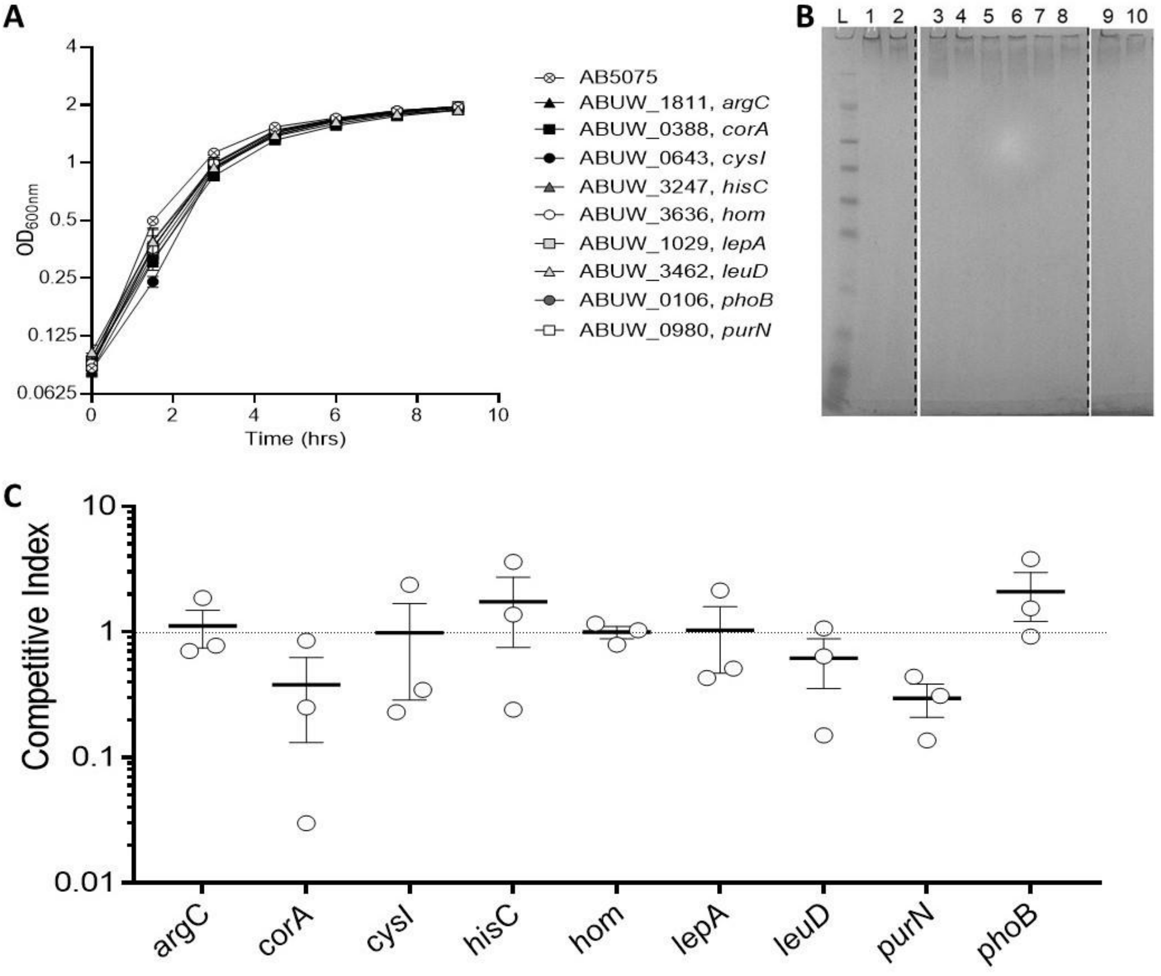

Supplementary Figure 6. *In vitro* validation of single mutant phenotypes. **(A)** Growth kinetics for individual transposon mutants under standard laboratory conditions, in LB at 37°C with aeration. All mutants have growth kinetics comparable with that of the parental strain *A. baumannii* AB5075-UW. Mean ± SEM were calculated from three independent experiments conducted on different days. **(B)** Alcian blue staining of capsular polysaccharide extracted from individual mutant strains, separated using 4–12% Bis-Tris SDS-PAGE. L = See Blue Plus2 Prestained Standard (Invitrogen), 1. AB5075-UW wild-type, 2. *argC,* 3. *corA,* 4. *cysI,* 5. *hisC,* 6. *hom,* 7. *lepA,* 8. *leuD*, 9. *phoB*, 10. *purN.* Varying degrees of capsule and PNAG are observed in samples isolated from various transposon mutants. **(C)** *In vitro* competitive indices for each mutant when grown in competition with the parental strain *A. baumannii* AB5075-UW under

standard laboratory conditions, in LB media at 37°C with aeration. Competitive index of < 1 indicates a fitness defect.

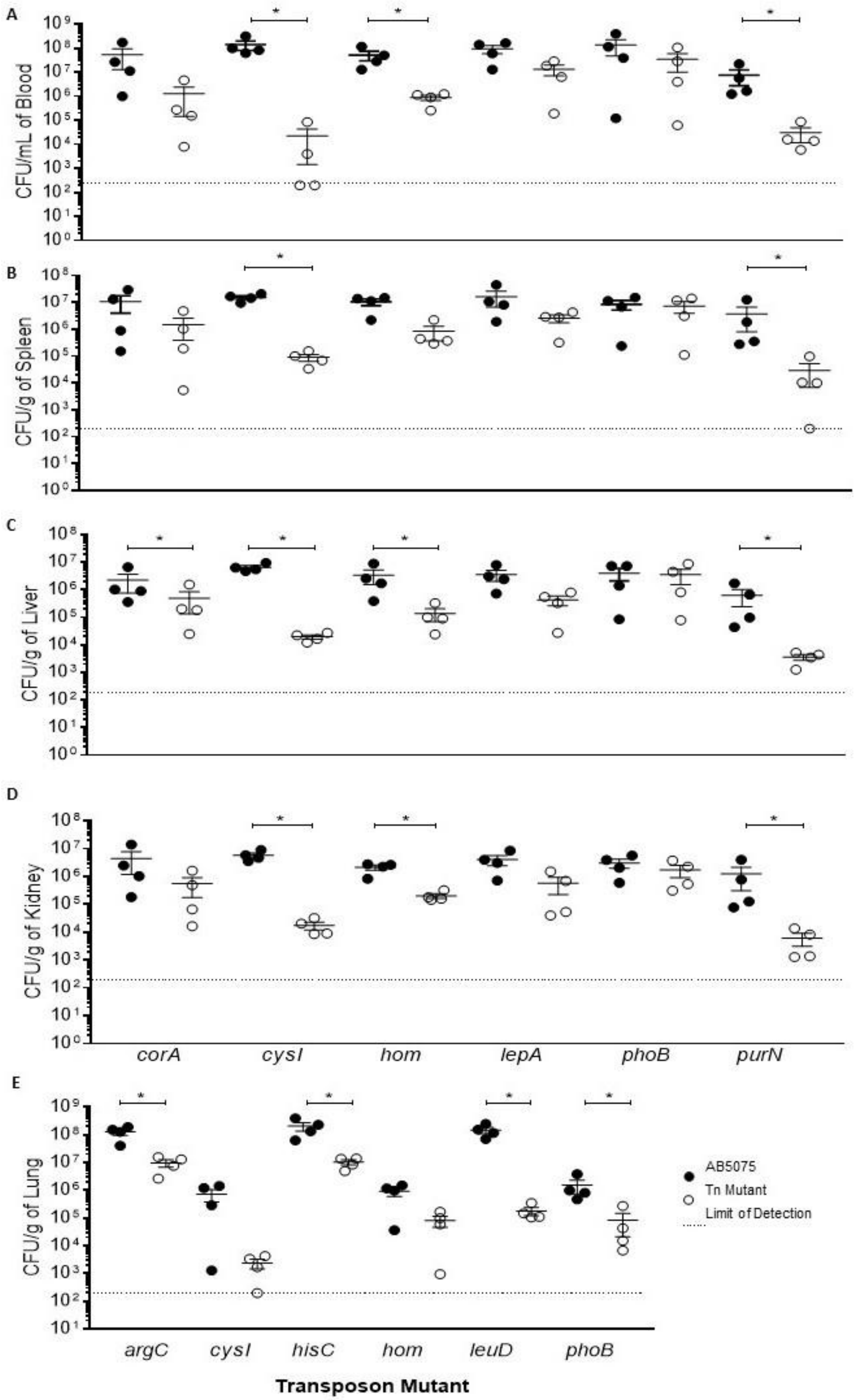
A
CFU/mL of Blood
B
CFU/g of Spleen
C
CFU/g of Liver
D
CFU/g of Kidney
corA
cysI
hom
lepA
phoB
purN
E
CFU/g of Lung
argC
cysI
hisC
hom
leuD
phoB
Transposon Mutant
AB5075
Tn Mutant
Limit of Detection

Supplementary Figure 7. *In vivo* validation of mutants with attenuated fitness during intraperitoneal and intranasal infections. Recovered CFU per gram of tissue or mL of **(A)** blood, **(B)** spleen, **(C)** liver, **(D)** kidneys, and **(E)** lungs, eight hours post competitive infection, whereby black circles represent the wild-type *A. baumannii* AB5075-UW and white circles show the corresponding burden of transposon mutant. Four mice were included per group and experiments conducted on two separate days for each infection route. Mean ± SEM are represented by the horizontal and vertical lines. Statistically significant differences in bacterial colonization were determined using Mann-Whitney, * P = <0.05.
